# Introducing the Concept of Exercise Holidays for Human Spaceflight - What Can We Learn From the Recovery of Bed Rest Passive Control Groups

**DOI:** 10.3389/fphys.2022.898430

**Published:** 2022-07-04

**Authors:** Robert Ekman, David A. Green, Jonathon P. R. Scott, Roger Huerta Lluch, Tobias Weber, Nolan Herssens

**Affiliations:** ^1^ Riga Stradins University, Faculty of Medicine, Riga, Latvia; ^2^ Space Medicine Team (HRE-OM), European Astronaut Centre, European Space Agency, Cologne, Germany; ^3^ Centre of Human and Applied Physiological Sciences, King’s College London, London, United Kingdom; ^4^ KBR GmbH, Cologne, Germany; ^5^ Institut Médecine Physiologie Spatiale (MEDES), Toulouse, France; ^6^ MOVANT, Department of Rehabilitation Sciences and Physiotherapy, Faculty of Medicine and Health Sciences, University of Antwerp, Antwerp, Belgium

**Keywords:** microgravity, spaceflight, deconditioning, astronaut, countermeasures

## Abstract

In an attempt to counteract microgravity-induced deconditioning during spaceflight, exercise has been performed in various forms on the International Space Station (ISS). Despite significant consumption of time and resources by daily exercise, including around one third of astronauts’ energy expenditure, deconditioning—to variable extents—are observed. However, in future Artemis/Lunar Gateway missions, greater constraints will mean that the current high volume and diversity of ISS in-flight exercise will be impractical. Thus, investigating both more *effective* and *efficient* multi-systems countermeasure approaches taking into account the novel mission profiles and the associated health and safety risks will be required, while also reducing resource requirements. One potential approach is to reduce mission exercise volume by the introduction of exercise-free periods, or “*exercise holidays*”. Thus, we hypothesise that by evaluating the ‘recovery’ of the no-intervention control group of head-down-tilt bed rest (HDTBR) campaigns of differing durations, we may be able to define the relationship between unloading duration and the dynamics of functional recovery—of interest to future spaceflight operations within and beyond Low Earth Orbit (LEO)—including preliminary evaluation of the concept of exercise holidays. Hence, the aim of this literature study is to collect and investigate the post-HDTBR recovery dynamics of current operationally relevant anthropometric outcomes and physiological systems (skeletal, muscular, and cardiovascular) of the passive control groups of HDTBR campaigns, mimicking a period of ‘exercise holidays’, thereby providing a preliminary evaluation of the concept of ‘exercise holidays’ for spaceflight, within and beyond LEO. The main findings were that, although a high degree of paucity and inconsistency of reported recovery data is present within the 18 included studies, data suggests that recovery of current operationally relevant outcomes following HDTBR without exercise—and even without targeted rehabilitation during the recovery period—could be timely and does not lead to persistent decrements differing from those experienced following spaceflight. Thus, evaluation of potential exercise holidays concepts within future HDTBR campaigns is warranted, filling current knowledge gaps prior to its potential implementation in human spaceflight exploration missions.

## 1 Introduction

Spaceflight is associated with anthropometric adaptations such as loss of body mass (Matsumoto et al., 2011), stature increments (Green and Scott, 2018) and deconditioning of physiological systems including musculoskeletal (Trappe et al., 2009; Stavnichuk et al., 2020) and cardiopulmonary deconditioning (Charles and Lathers, 1991; Hargens and Richardson, 2009). To counteract microgravity-induced deconditioning, exercise in various forms has been performed since early space missions and has evolved significantly over the years (Hayes, 2015; Scott et al., 2019). Current exercise prescriptions for ESA astronauts on-board the International Space Station (ISS) consist of approximately 90 min concurrent aerobic and resistive exercise training per day throughout long-duration missions, involving use of a resistive exercise device (ARED), a treadmill (T2) and a cycle ergometer (CEVIS) (Petersen et al., 2016). As a result, around one third of the astronauts’ daily energy expenditure is spent on exercise (Laurens et al., 2019; Scott et al., 2020). Despite this, multi-system physiological deconditioning—albeit to variable extents—is still observed in most long-duration ISS crew (Weber et al., 2020; Scott et al., 2021).

With entirely new mission profiles on the horizon (e.g., Artemis and Lunar Gateway), where microgravity exposure will be significantly shorter, but where crew will be exposed to Lunar hypogravity upon landing on the Lunar surface (NASA, 2020), needs and requirements for in-flight exercise countermeasures will likely change significantly, driven by the novel mission profiles and associated health and safety risks. This could imply that for Lunar gateway missions with Lunar surface EVAs after prolonged (30–90 days) exposure to microgravity in Lunar orbit, primary needs and requirements of the countermeasure programmes may not need to focus on maintaining bone mineral density, muscle strength and VO2max as is the case in current long-duration mission profiles. However, a unique and critical period in these missions will be the transition from prolonged exposure to microgravity, to hypogravity on the Lunar surface. Both Miller et al. (2018) and Mulavara et al. (2018) reported significantly worse performances of functional tasks (e.g., seat egress and walk, recovery from fall, jump down) and sensorimotor tests (e.g., dynamic posturography, tandem walk) following long-duration spaceflight with extensive daily exercise regimens. Thus, recovery of orthostatic tolerance, postural stability, spatial orientation, and balance will likely be of greater importance to assure crew safety and mission success as is currently the case. Therefore, definition of future in-flight countermeasure programmes will most likely benefit from shifting the focus from current operationally relevant parameters for long-duration spaceflight (i.e., skeletal, muscular, and cardiovascular) to those more relevant to the new mission profiles involving Lunar surface EVAs. Additionally, vehicle constraints will also mean that the currently prescribed high volume-high load exercise with a great energy expenditure and diversity of ISS in-flight exercise currently prescribed might not be appropriate (Laurens et al., 2019). Optimization of exercise programmes could also reduce the metabolic cost, and thus associated energy expenditure, thereby reducing food, water and respiratory gas (i.e., oxygen provision and carbon dioxide removal) requirements, which would be highly advantageous since re-supply opportunities will be greatly reduced, or impossible (Drake et al., 2010). One potential approach to reduce overall exercise volume and associated energy expenditure is the introduction of exercise-free periods, or “*exercise holidays*”, throughout—a part of—the duration of the space mission.

Exercise holidays are commonly prescribed to elite athletes, including offseason breaks as part of training periodization that seeks to facilitate optimal performance during specific periods (Lorenz et al., 2010). During periodization, training variables such as type, load, sets and within set repetitions are manipulated to maximize appropriate training adaptations, whilst attempting to minimize excessive fatigue, and or injury risk (Buford et al., 2007; Lorenz et al., 2010). Hence, athletes may be prescribed periods where exercise volume and intensity are significantly reduced or even minimal (Lorenz et al., 2010).

Translating this to the context of spaceflight, crewmembers would thus be prescribed periods without in-flight exercise countermeasures—increasing the time to be spent on scientific research, maintenance, or extravehicular activities—and periods with in-flight exercise countermeasures, tasked to optimize functionality in-flight, during landing or the immediate post-flight period. However, they do not seek to optimize athletic performance, but rather maintain health, wellbeing and functionality, in particular upon landing when astronauts are exposed to hypogravity on the Lunar surface, or re-exposed to Earth’s gravity in a state of microgravity-induced deconditioning.

In fact, astronaut gravitational unloading is more akin to bed-bound patients, such as those admitted to intensive care. Such patients experience rapid and profound musculoskeletal (Puthucheary et al., 2010) and cardiopulmonary deconditioning (Benington et al., 2012) leading to a protracted impairment of everyday activities (Svenningsen et al., 2017). As a result, intensive rehabilitation is required to promote performance of everyday activities, resumption of independence and the improvement of quality of life (Denehy and Elliott, 2012; Svenningsen et al., 2017).

The most commonly employed ground-based analogue is long term six-degree head-down-tilt bed rest (HDTBR) which mimics many of the physiological effects associated with long-duration space missions (Hargens and Vico, 2016). HDTBR studies have the advantage of being able to study larger sample sizes, allow better standardisation (e.g., fixed daily routine for all participants), and to minimise some of the potential confounding factors associated with spaceflight (e.g., space radiation) (Kakurin et al., 1976; Regnard et al., 2001; Winnard et al., 2017). HDTBR studies of differing durations have been performed, reporting broadly similar changes in anthropometric (e.g., mass loss (Matsumoto et al., 2011)), skeletal (e.g., reduced bone mineral density (Baecker et al., 2003) and altered bone architecture (Spector et al., 2009)), muscular (e.g., loss of muscle mass (Droppert, 1993)), and cardiovascular parameters (e.g., reduced cardiac output (Arbeille et al., 2001)), as those observed following spaceflight. As a result, HDTBR participants also require a period of rehabilitation (Winnard et al., 2019).

Thus, improving the understanding of induced de-conditioning, but mainly the dynamics of recovery of passive control groups of HDTBR campaigns is essential. Such knowledge is critical for defining, evaluating, and optimizing in-flight exercise countermeasure prescriptions of future space exploration missions, but may also facilitate evaluation of the concept of exercise holidays.

However, to this date, the post-HDTBR recovery period has received relatively little attention. In fact, despite numerous HDTBR studies being performed, there is still no agreement on the approach to rehabilitation (Winnard et al., 2017). Furthermore, very few HDTBR participants have received an individualized rehabilitation programme similar to that provided to astronauts (Petersen et al., 2016, Petersen et al., 2017). Indeed, the lack of attention paid to the post-HDTBR period was highlighted by Greenleaf and Quach (2003), having reviewed, at that time, 157 published HDTBR studies. Greenleaf and Quach also highlighted a single study that evaluated an exercise protocol consisting of supine treadmill walking and a cycle ergometer that was instigated at day 140 of a HDTBR study, reporting that various musculoskeletal and cardiovascular parameters returned to baseline by day 240 of continuing HDTBR (Grigoriev et al., 1992). This data suggests that the concept of an exercise holiday may hold promise—but is insufficient on its own. To further explore this, we hypothesise that by evaluating the recovery of the passive control groups of HDTBR campaigns of differing durations we may be able to gain insights into the dynamics of functional recovery following a period of simulated exercise holidays.

Thus, the aim of this literature study is to, for the first time, collect and investigate the post-HDTBR recovery dynamics of current operationally relevant anthropometric outcomes and physiological systems (skeletal, muscular, and cardiovascular) of the passive control groups of HDTBR campaigns, mimicking a period of exercise holidays, thereby providing a preliminary evaluation of the concept of ‘exercise holidays’ for spaceflight, within and beyond Low Earth Orbit (LEO).

## 2 Materials and Methods

### 2.1 Data Sources and Searches

An initial systematic search was performed based on that reported by (Fiebig et al., 2018) that used Boolean search strings based on three overarching categories (“microgravity”, “countermeasures”, and “operationally relevant outcome parameters”), as defined by ESA’s Space Medicine Team. This search, performed on 18 June 2021, evaluated the various databases: Pubmed, Web of Science, Cochrane Collaboration Library, Institute of Electrical and Electronics Engineers database as well as ESA’s “Erasmus”, the National Aeronautics and Space Administration’s (NASA) “Life Science Data Archive” and “Technical Reports Server” and the German Aerospace Centre’s (DLR) database “elib” for relevant studies published in English.

Additionally, a second search was performed on 7 July 2021 in Pubmed only, based on the “microgravity” and “operationally relevant outcome parameters” categories to ensure no studies were excluded that did not include a countermeasure intervention.

Results of both searches were combined, and duplicate records removed to yield a single file used for study selection (see *Supplementary Table S1*).

### 2.2 Study Selection

Relevant studies were identified using predefined selection criteria according to the Population Intervention Comparison Outcomes Study design (PICOS) methods:

1) **Population—**Healthy adult female and/or male bed rest participants (≥18 years old).
2) **Interventions—**Studies utilizing six-degree head-down tilt bed rest with a minimum duration of 5 days—in accordance with the categories for bed rest study duration described by Sundblad and Orlov (2014)—and at least two follow-up evaluations during the post-bed rest (recovery) period.
3) **Control Conditions—**Only bed rest participants that were assigned to a passive/no intervention/placebo control condition were included in this review. Data from participants assigned to an exercise, nutrition, or any other intervention were not extracted.
4) **Outcomes**—Only studies containing outcomes considered to be “operationally relevant” were included. Outcome parameters within the categories of interest (see below) were defined as “operationally relevant” by members of ESA’s Space Medicine Team who performed a scoping exercise based on parameters reported in papers extracted by Fiebig et al. (2018) where relevance was defined as:
5) “Parameters having a direct impact on physical performance in space and after landing, and/or that would jeopardise nominal mission performance when deteriorated.”
6) **Study Designs**—Randomised controlled trials (RCT) and controlled clinical trials (CT) were included.

Phase 1 involved several independent reviewers (RE, TW, NH, RHL, DG) independently (blinded) applying the selection criteria on titles and abstracts via the Rayyan Web Application (Ouzzani et al., 2016). Phase 2 involved blinded screening of the full-text resources, based on the same pre-defined selection criteria.

### 2.3 Data Extraction and Quality Assessment

#### 2.3.1 Data Extraction

If the study was eligible, the following data were extracted:

1) **General Population Characteristics**—Number of participants, sex distribution, mean, standard deviation (SD) and range of age (years), body height (centimetres) and body mass (kg).
2) **Characteristics of the Six Degree Head Down Bed Rest Intervention—**Number of bed rest days, diet, daily routine, standardization of bed-rest phases (e.g., same baseline data collection, same bed-rest time), sunlight exposure.
3) **Characteristics of the Recovery Period**—Number of days of follow-up, time-points of measurements during recovery period, standardization of recovery period (e.g., controlled recovery phases and conditions).
4) **Reported Outcome Parameters—**Numeric values (Mean and SD/standard error (SE); Median and Interquartile Range; % change from baseline with SD) for each relevant parameter at baseline and at each time-point during recovery were extracted. Each parameter was classified under one of the following categories: “Anthropometric Outcomes”, “Skeletal System”, “Muscular System” and “Cardiovascular System”. For a full overview of all extracted parameters, see *Supplementary Material 1* *—Operationally Relevant Outcome Parameters.*

As adaptation of physiological s-ystems during the recovery period were largely of secondary importance in the majority of included HDTBR studies, recovery data was extrapolated where appropriate from tables and figures. Extrapolation of data from figures was performed with WebPlotDigitizer (version 4.5; California, United States) software, which has been shown to yield reliable and valid data (Drevon et al., 2016).

#### 2.3.2 Quality Assessment

Quality appraisal of the methodology of the included bed rest studies was assessed using the AMSRG tool (Winnard and Nasser, 2017). This purpose-built tool uses eight criteria to detail how similar the conditions of the ground-based analogue are compared with actual spaceflight, thereby assessing the ability to simulate the physiological effects of a prolonged exposure to microgravity: 1) Number of bed rest days stated; 2) six degrees head down tilt; 3) individualized and controlled diet; 4) set daily routine with fixed wake/sleep time; 5) bed rest phases standardised for all participants; 6) uninterrupted bed rest except for test condition; 7) sunlight exposure prohibited; 8) all measures taken at the same day and time.

Each study was assessed against each criterion, whether it was met “Y”; not met “N”; or whether it was unclear/information was lacking “?“. All criteria which were met are ascribed a value of 1 and summed to yield a total score: ranging from 0 (poor) to 8 (excellent).

### 2.4 Data Analysis

To determine whether an outcome parameter recovered following the HDTBR period, standardized mean differences (Hedges *g*; mean and 95% confidence interval) (Cumming, 2012; Lakens, 2013) were calculated from the reported raw pre- and post-HDTBR mean and SD values (*Supplementary Material 2* *—Statistical Calculations*).

A given parameter was deemed to have “recovered” during the recovery period according to the Westlake’s Confidence Interval procedure (Seaman and Serlin, 1998) which evaluates mean equivalence using a confidence interval (e.g., 95% CI of Hedges *g*) for the difference between two means. Upper (0.49) and lower (-0.49) equivalency bounds of interest, corresponding to the limit of a small effect size (Sawilowsky, 2009), were then determined. When combined with the 95% CI of the calculated Hedges *g*, three scenarios are possible (Seaman and Serlin, 1998):

1) The 95% CI falls completely outside the set equivalency bounds, thus it cannot be concluded that the difference between means is trivial, hence no evidence of recovery is observed.
2) The 95% CI falls partially within the set equivalency bounds, the results are inconclusive as the 95% CI included both trivial and non-trivial mean differences, hence weak evidence of recovery is observed.
3) The 95% CI falls completely within the set equivalency bounds, the 95% CI reveals a trivial difference, there is practical equivalence, hence strong evidence of recovery is observed.

Using the Confidence Interval approach has two main advantages (Quertemont, 2011). Firstly, the underlying reasoning is easy to understand, i.e., if both limits of the 95% CI are within the predetermined threshold values it can be concluded that there is no effect of practical importance. Secondly, there is no need to agree on a precise value of the threshold for a minimal effect size allowing interpretation of whether the interval limits are sufficiently narrow to be of no practical significance.

If only the percentage change from baseline data was reported for the recovery time-points, values could not be transformed into raw mean and SDs and were thus excluded from Hedges *g* calculations. In this case, vote counting based on direction of effects was used to synthesize such results (Higgins et al., 2021). For each study, the effect was categorized as ‘Returned to Baseline’ or ‘Not Returned to Baseline’. An outcome was deemed to have ‘Returned to Baseline’ whenever the Mean % Change from Baseline was equal to 0%, or when a negative Mean % Change became positive, or vice versa. An outcome was deemed to have ‘Not Returned to Baseline’ when the Mean % Change from Baseline remained negative, or positive. The number of effects that Returned to Baseline were then compared with the number that was deemed to have Not Returned to Baseline and were synthesized as the ratio between the number of effects that were deemed to have Returned to Baseline, and the total number of effects reported for that particular outcome.

## 3 Results

**FIGURE 1:**
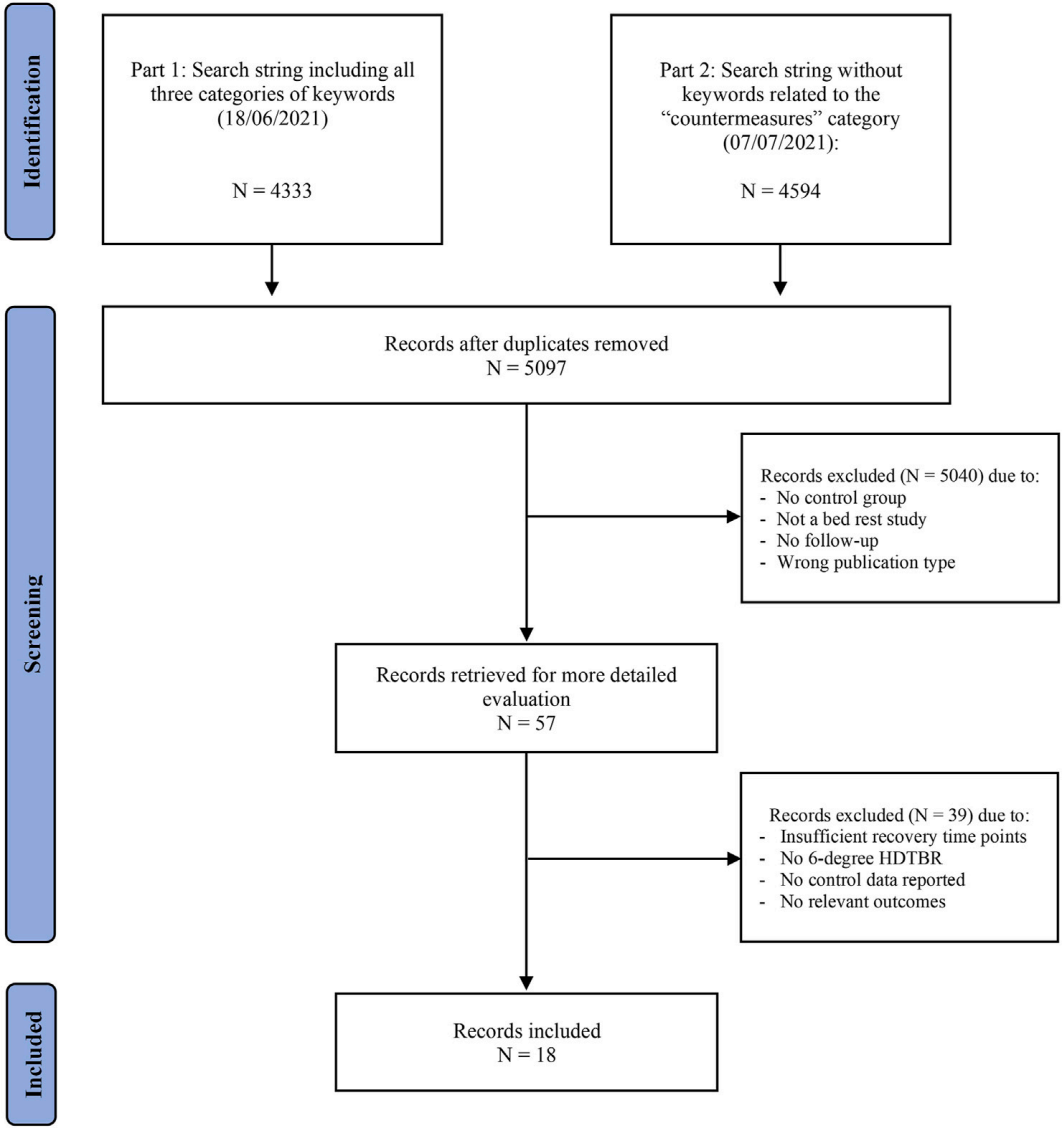
Flowchart of the selection process.

The initial search query generated 5,097 unique hits. After screening, 18 studies (Stegemann et al., 1985; Convertino et al., 1990; Beck et al., 1992; Schulz et al., 1992; Samel et al., 1993; Ferretti et al., 2001; Alkner and Tesch, 2004; Linnarsson et al., 2006; Rittweger et al., 2007; Rittweger and Felsenberg, 2009; Belavý et al., 2011a; Beller et al., 2011; Liu et al., 2015; Rittweger et al., 2015; Alkner et al., 2016; Westby et al., 2016; Belavý et al., 2017; Kramer et al., 2017) met the selection criteria from which data were extracted (Figure 1).

### 3.1 Quality Assessment—Bed Rest Methodology

Total AMSRG scores of the included studies ranged between 4 and 7 out of 8 with a mean score of 5.8 ± 1.0 (Table 1). All included studies failed to provide clarity on whether sunlight exposure was prohibited and whether participants were supplemented with vitamin D. Only five studies (36%) (Convertino et al., 1990; Samel et al., 1993; Belavý et al., 2011a; Rittweger et al., 2015; Kramer et al., 2017) reported whether the daily routine was fixed, additionally, information regarding individualized and controlled diet was absent in five (35%) (Stegemann et al., 1985; Ferretti et al., 2001; Alkner and Tesch, 2004; Linnarsson et al., 2006; Alkner et al., 2016) of the included studies. Two studies (Alkner and Tesch, 2004; Alkner et al., 2016) failed to address whether the head-down tilt was maintained throughout the entire bedrest period, whilst one study (Liu et al., 2015) reported that participants were allowed to use the bathroom for 5–10 min a day.

**TABLE 1:** Quality appraisal of bed rest method to simulate microgravity.

Author	Number of BR days	6° Head down Tilt	Individualized and Controlled Diet	Set Daily Routine with Fixed Wake/Sleep Time	BR Phases Standardized for all Participants	Uninterrupted BR except for Test Condition	Sunlight Exposure Prohibited	All Measurements Taken Same day and Time	*Total score*
*Short duration HDTBR (5—14 days)*									
Beck et al. (1992)	10	Y	Y	?	Y	Y	?	Y	6
Rittweger et al. (2015)	5	Y	Y	Y	Y	Y	?	Y	7
Samel et al. (1993)	7	Y	Y	Y	Y	Y	?	Y	7
Schulz et al. (1992)	10	Y	Y	?	Y	Y	?	Y	6
Stegemann et al. (1985)	7	Y	?	?	Y	Y	?	Y	5
*Medium duration HDTBR (15—59 days)*									
Convertino et al. (1990)	30	Y	Y	Y	Y	Y	?	Y	7
Ferretti et al. (2001)	42	Y	?	?	Y	Y	?	Y	5
*Long duration HDTBR (≥60 days)*									
Alkner and Tesch (2004)	90	Y	?	?	Y	?	?	Y	4
Alkner et al. (2016)	90	Y	?	?	Y	?	?	Y	4
Belavý et al. (2011a)	60	Y	Y	Y	Y	Y	?	Y	7
Belavý et al. (2017)	90	Y	Y	?	Y	Y	?	Y	6
Beller et al. (2011)	60	Y	Y	?	Y	Y	?	Y	6
Kramer et al. (2017)	60	Y	Y	Y	Y	Y	?	Y	7
Linnarsson et al. (2006)	120	Y	?	?	Y	Y	?	Y	5
Liu et al. (2015)	60	Y	Y	?	Y	N	?	Y	5
Rittweger et al. (2007)	90	Y	Y	?	Y	Y	?	Y	6
Rittweger and Felsenberg (2009)	90	Y	Y	?	Y	Y	?	Y	6
Westby et al. (2016)	60	Y	Y	?	Y	Y	?	Y	6
*Average*									5.8
*SD*									1.0

**Note.** This tool allows to assess how well bed rest studies have been conducted to simulate actual human spaceflight developed by (Winnard et al., 2017; Winnard and Nasser, 2017). The higher the total score, the better the quality and the greater the transferability to human spaceflight; BR: bed rest; Y: yes, criteria is met; N: no, criteria is not met; ? Unclear/information is lacking.

### 3.2 Study Characteristics

From the 18 studies, selected data were extracted from 169 participants (11 females, 6.5%) within control/no-countermeasure groups with ages ranging between 20 (Stegemann et al., 1985) and 45 (Convertino et al., 1990) years old. The duration of −6° HDTBR ranged between five (Rittweger et al., 2015) and 120 days (Linnarsson et al., 2006), and included five studies of short duration (5–14 days) HDTBR (Stegemann et al., 1985; Beck et al., 1992; Schulz et al., 1992; Samel et al., 1993; Rittweger et al., 2015), two studies of medium (15–59 days) duration (Convertino et al., 1990; Ferretti et al., 2001), and 11 studies of long duration (≥60 days) HDTBR (Alkner and Tesch, 2004; Linnarsson et al., 2006; Rittweger et al., 2007; Rittweger and Felsenberg, 2009; Belavý et al., 2011a; Beller et al., 2011; Liu et al., 2015; Alkner et al., 2016; Westby et al., 2016; Belavý et al., 2017; Kramer et al., 2017), as categorized by Sundblad and Orlov (2014). Reported recovery periods lasted between 2 (Samel et al., 1993), and 360 days (Rittweger and Felsenberg, 2009; Belavý et al., 2017) (Table 2).

**TABLE 2:** Characteristics of the individual studies.

Author	Bedrest campaign	# Days bed rest	# Days recovery period	Study Sample Characteristics									Space agencies involved	Location - setting
				*# Subjects*	*# Females*	*Age (years)*			*Body length (cm)*		*Body Weight (kg)*			
						Mean	SD	Min-Max	Mean	SD	Means	SD		
*Short duration HDTBR (5—14 days)*														
Beck et al. (1992)	HDT′88 study	10	8	6	0	26	4.4	21–34	176	5	72	12.4	DLR, NASA	Germany - DLR
Rittweger et al. (2015)	BRAG1 study	5	5	11	0	34	7	22–42	179	7	76	6	ESA	France - MEDES Facilities
Samel et al. (1993)		7	2	8	0	23.9	2	21–27					DLR	Germany - DLR
Schulz et al. (1992)	HDT′88 study	10	8	6	0	26	4.4	21–34	176	5	72	12.4	DLR, NASA	Germany - DLR
Stegemann et al. (1985)		7	5	6	0	23.3	2.81	20–28	180.7	4.97	73.5	7.6		Germany
*Medium duration HDTBR (15—59 days)*														
Convertino et al. (1990)		30	30	11	0	38	6.6	30–45	179	2	79	2	NASA	US - NASA-Ames Research Center Human Research Facility
Ferretti et al. (2001)	HDT 94 BR project	42	48	7	0	28	1		176	1	74.7	8.8	ESA	France - MEDES Facilities
*Long duration HDTBR (≥60 days)*														
Alkner and Tesch (2004)		90	11	9	0	32	4		173	3	72	5		France - MEDES Facilities
Alkner et al. (2016)		90	11	9	0	32	4		173	3	72	5		France - MEDES Facilities
Belavý et al. (2011a)	2nd Berlin Bed Rest Study	60	90	9	0	33.1	7.8		181.3	6	80.6	5.2	ESA, DLR	Germany - Charite Campus Bejamin Franklin (Berlin)
Belavý et al. (2017)	LTBR study	90	360	16	0	32.5	3.4		174	4	70.3	6.1	ESA, CNES, NASDA	France - MEDES Facilities
Beller et al. (2011)	WISE-2005	60	20	8	8	34.4	3.8		162.8	6.2	56.5	3.3	ESA, NASA, CSA, DLR, CNES	France - MEDES Facilities
Kramer et al. (2017)	Cologne RSL study	60	15	11	0	28	6		181	5	76	8	ESA, DLR	Germany - Envihab facility (DLR)
Linnarsson et al. (2006)		120	15	6	0	31		23–42	181		80		ESA	Russia - Institute for Biomedical Problems, Moscow
Liu et al. (2015)		60	15	14	0	30	1		169	1				China - Bed Rest Study Lab - China Astronaut Research and Training Center
Rittweger et al. (2007)	LTBR study	90	180	16	0	32.5	3.4		174.2	3.9	71.4	6.7	ESA, CNES, NASDA	France - MEDES Facilities
Rittweger and Felsenberg (2009)	LTBR study	90	360	9	0	31.9	3.6	26–37	173.4	3	71.7	5.4	ESA, CNES, NASDA	France - MEDES Facilities
Westby et al. (2016)		60	14	7	3	36	8				72.5	2.8	NASA	US - Flight Analog Research Unit

### 3.3 Recovery of ‘Anthropometrics Outcomes’

**FIGURE 2:**
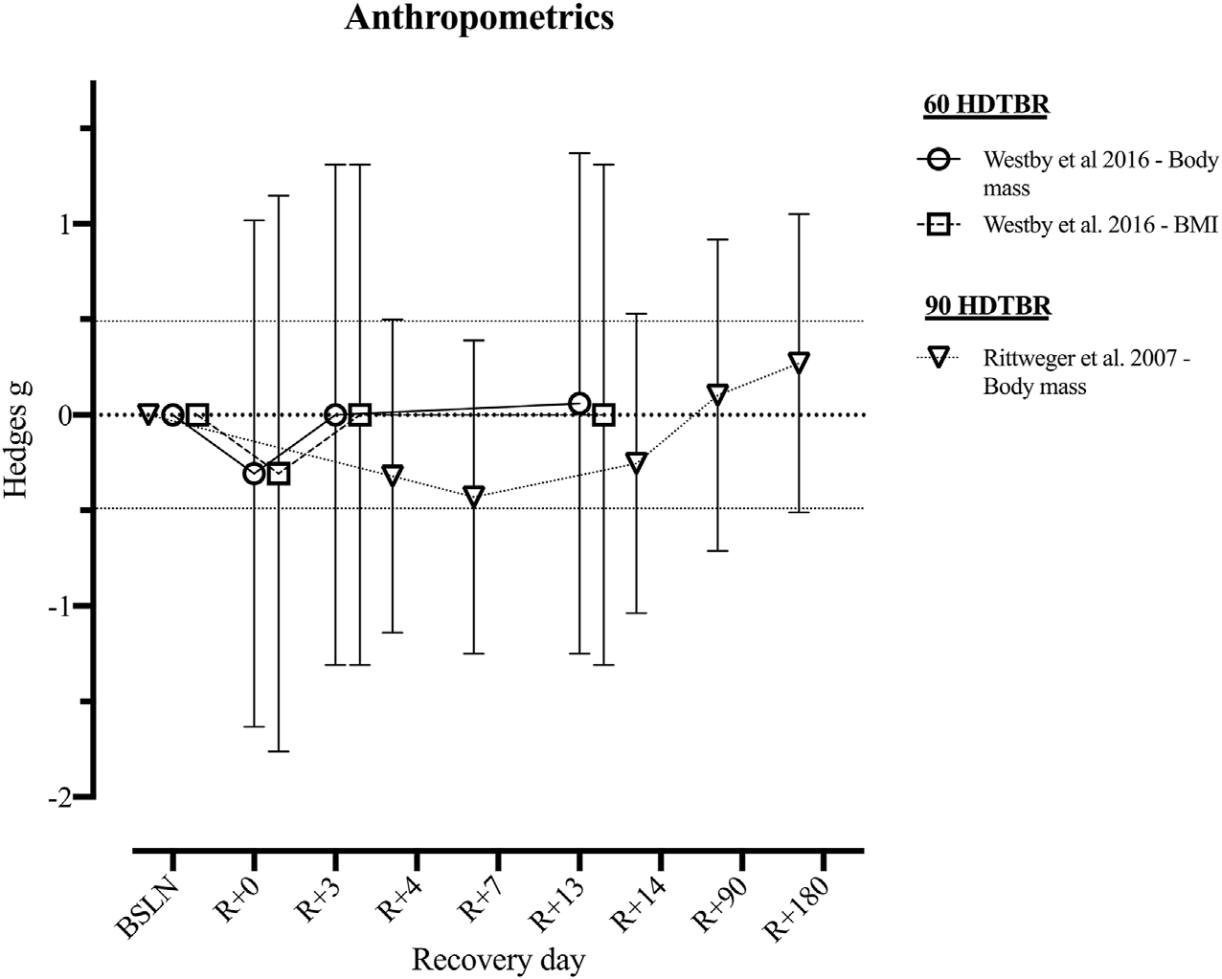
Visualisation of the recovery of outcomes related to ‘*Anthropometrics Outcomes*’ after a period of head-down-tilt bed rest, displayed as Hedges *g* with 95% Confidence Interval. To determine whether a particular outcome could be deemed as “recovered” during the recorded recovery period, the Westlake’s Confidence Interval Procedure (Seaman and Serlin, 1998) was used. This procedure tests for equivalence between two means using a confidence interval. To do so, Upper (0.49) and Lower (−0.49) equivalency bounds of interest were determined, corresponding to the limit of a small effect size. When combined with the 95% Confidence Interval of the Hedges *g*, three scenarios are possible: 1) *No evidence of recovery*: It cannot be concluded that the difference between means is trivial as the 95% Confidence Interval falls completely outside the set equivalency bounds; 2) *Weak evidence of recovery*: The results are inconclusive as the 95% Confidence Interval partially falls within the set equivalency bounds, thus including both trivial and non-trivial differences. The dotted lines at 0.49 and −0.49 represent the upper and lower equivalency bounds; 3) *Strong evidence of recovery:* There is practical equivalence as the 95% Confidence Interval falls completely within set equivalency bounds; 60 HDTBR: Included studies implementing a head-down-tilt bed rest period of 60 days (Westby et al., 2016); 90 HDTBR: Included studies implementing a head-down-tilt bed rest period of 90 days (Rittweger et al., 2007); R+…: Respective recovery day; BMI: Body Mass Index.

None of the short and medium duration HDTBR studies reported on the recovery of anthropometric outcomes. Two long duration HDTBR studies (Rittweger et al., 2007; Westby et al., 2016) provided sufficient data to calculate effect sizes for outcomes related to anthropometric outcomes (Figure 2 and *Supplementary Table S2*). Following 60 days of HDTBR, mean body weight and BMI returned to baseline values by R+3, with Hedges *g* of 0.00 [−1.31; 1.31] (Westby et al., 2016). Following a 90-days HDTBR, body weight increased during the recovery period and surpassed the baseline value by R+90 (*g* = 0.10 [−0.71; 0.92]) (Rittweger et al., 2007).

One long duration HDTBR study (Kramer et al., 2017) reported the percentage change from baseline for total body mass, fat mass and lean mass (Table 3), indicating an increase in total body mass and lean mass from R+7 to R+14, while fat mass decreased. Yet, none of the outcomes returned to baseline within the recorded recovery period.

**TABLE 3:** % Change with SD of outcome parameters related to *‘Anthropometric Outcomes*’.

	Recovery timepoint	Author	Returned to baseline? = Y
		Kramer et al. (2017)	
		*60 days HDTBR*	
*Body Mass*			
	**R+7**	−2.23 (1.88)^a^	
	*Returned to Baseline? (Y/N)*	N	0/1
	**R+14**	−1.80 (1.69)	
	*Returned to Baseline? (Y/N)*	N	0/1
*Fat Mass*			
	**R+7**	0.34 (8.80) ^a^	
	*Returned to Baseline? (Y/N)*	N	0/1
	**R+14**	0.17 (7.92) ^a^	
	*Returned to Baseline? (Y/N)*	N	0/1
*Lean Mass*			
	**R+7**	−2.55 (3.10) ^a^	
	*Returned to Baseline? (Y/N)*	N	0/1
	**R+14**	−1.47 (2.64) ^a^	
	*Returned to Baseline? (Y/N)*	N	0/1

**Notes.** data of outcome parameters related to anthropometric outcomes, reported as % change from baseline were extracted and displayed without any alterations for each reported recovery timepoint following a period of 6-degree-head-down-tilt bed rest. For each recovery timepoint the effect was categorize as “Returned to baseline” or “Not returned to baseline”. Returned to Baseline? = Y: Whenever the mean % change equals 0% or reverts from + to—/– to +; Returned to Baseline? = N: Whenever the mean % change remains + or –.All values are displayed as Mean % Change from Baseline (SD); HDTBR: 6-degree-head-down-tilt bed rest; Y: yes; N: no.

a Data extracted from figure using WebPlotDigitizer.

### 3.4 Recovery of the ‘Skeletal System’

None of the included studies reported sufficient information to calculate effect sizes of operationally relevant outcomes related to the skeletal system.

Three long duration HDTBR studies (Rittweger and Felsenberg, 2009; Belavý et al., 2011a; Beller et al., 2011) provided information on the percentage change from baseline for outcomes related to bone mineral content (BMC) and bone mineral density (BMD) (Table 4). Following a 60-days HDTBR (Belavý et al., 2011a), lumbar spine BMC returned to baseline values by R+14, while total body BMC, legs BMC and distal tibia BMC increased, and trunk BMC decreased between R+3 and R+90, but did not return to baseline. For BMD of hip and distal tibia, Beller et al. (2011) reported an increase in BMD, yet values did not reach baseline values at R+360 following a 60-days HDTBR period. Lumbar spine BMD did however show an increase—compared to baseline—in the period of R+4 to R+180 yet was decreased at R+360. Rittweger and Felsenberg (2009) reported, after a 90-days HDTBR period, an increase of both the proximal (4%) and distal (66%) tibia BMC between R+4 and R+360, while only the distal tibia BMC surpassed the baseline values by R+360.

**TABLE 4:** % Change with SD of outcome parameters related to the *‘Skeletal System’*.

	Recovery timepoint	Author			Returned to baseline? = Y
		Belavý et al. (2011a)	Beller et al. (2011)	Rittweger and Felsenberg (2009)	
		*60 days HDTBR*	*60 days HDTBR*	*90 days HDTBR*	
*Lumbar Spine (L1-L4) BMC*					
	**R+3**	−1.30 (0.77)			
	*Returned to Baseline? (Y/N)*	N			0/1
	**R+14**	0.52 (0.85)			
	*Returned to Baseline? (Y/N)*	Y			1/1
	**R+30**	1.77 (0.87)			
	*Returned to Baseline? (Y/N)*	Y			1/1
	**R+90**	0.42 (0.89)			
	*Returned to Baseline? (Y/N)*	Y			1/1
*Total Body BMC*					
	**R+3**	−0.44 (0.25)			
	*Returned to Baseline? (Y/N)*	N			0/1
	**R+14**	−0.86 (0.25)			
	*Returned to Baseline? (Y/N)*	N			0/1
	**R+30**	−0.63 (0.21)			
	*Returned to Baseline? (Y/N)*	N			0/1
	**R+90**	−0.41 (0.19)			
	*Returned to Baseline? (Y/N)*	N			0/1
*Legs BMC*					
	**R+3**	−2.35 (0.43)			
	*Returned to Baseline? (Y/N)*	N			0/1
	**R+14**	−2.87 (0.38)			
	*Returned to Baseline? (Y/N)*	N			0/1
	**R+30**	−2.49 (0.40)			
	*Returned to Baseline? (Y/N)*	N			0/1
	**R+90**	−1.27 (0.47)			
	*Returned to Baseline? (Y/N)*	N			0/1
*Trunk BMC*					
	**R+3**	1.86 (0.77)			
	*Returned to Baseline? (Y/N)*	N			0/1
	**R+14**	1.87 (0.77)			
	*Returned to Baseline? (Y/N)*	N			0/1
	**R+30**	1.29 (0.83)			
	*Returned to Baseline? (Y/N)*	N			0/1
	**R+90**	0.80 (0.87)			
	*Returned to Baseline? (Y/N)*	N			0/1
*Tibia (4%) BMC*					
	**R+14**			−6.03 (1.64) ^a^	
	*Returned to Baseline? (Y/N)*			N	0/1
	**R+90**			−2.95 (0.70) ^a^	
	*Returned to Baseline? (Y/N)*			N	0/1
	**R+180**			−1.93 (0.49) ^a^	
	*Returned to Baseline? (Y/N)*			N	0/1
	**R+360**			−0.95 (0.42) ^a^	
	*Returned to Baseline? (Y/N)*			N	0/1
*Tibia (66%) BMC*					
	**R+3**	-2.07 (0.52) ^a^			
	*Returned to Baseline? (Y/N)*	N			0/1
	**R+14**			−1.97 (0.41) ^a^	
	*Returned to Baseline? (Y/N)*			N	0/1
	**R+15**	−2.10 (0.56) ^a^			
	*Returned to Baseline? (Y/N)*	N			0/1
	**R+30**	−2.23 (0.62) ^a^			
	*Returned to Baseline? (Y/N)*	N			0/1
	**R+90**	−1.59 (0.63) ^a^		−0.74 (0.24) ^a^	
	*Returned to Baseline? (Y/N)*	N		N	0/2
	**R+180**			−0.11 (0.19) ^a^	
	*Returned to Baseline? (Y/N)*			N	0/1
	**R+360**			0.14 (0.12) ^a^	
	*Returned to Baseline? (Y/N)*			Y	1/1
*Tibia (4%) BMD*					
	**R+3**		−3.13 (0.86)^b^		
	*Returned to Baseline? (Y/N)*		N		0/1
	**R+90**		−1.89 (0.78)^b^		
	*Returned to Baseline? (Y/N)*		N		0/1
	**R+180**		−1.65 (0.79)^b^		
	*Returned to Baseline? (Y/N)*		N		0/1
	**R+360**		−1.81 (0.82)^b^		
	*Returned to Baseline? (Y/N)*		N		0/1
*Hip BMD*					
	**R+3**		−3.50 (0.55)^b^		
	*Returned to Baseline? (Y/N)*		N		0/1
	**R+45**		−2.80 (0.77)^b^		
	*Returned to Baseline? (Y/N)*		N		0/1
	**R+90**		−2.46 (0.66)^b^		
	*Returned to Baseline? (Y/N)*		N		0/1
	**R+180**		−2.03 (0.94)^b^		
	*Returned to Baseline? (Y/N)*		N		0/1
	**R+360**		−0.52 (0.65)^b^		
	*Returned to Baseline? (Y/N)*		N		0/1
*Lumbar Spine (L1-L4) BMD*					
	**R+3**		0.44 (0.85)^b^		
	*Returned to Baseline? (Y/N)*		N		0/1
	**R+45**		0.92 (0.82)^b^		
	*Returned to Baseline? (Y/N)*		N		0/1
	**R+90**		0.14 (0.75)^b^		
	*Returned to Baseline? (Y/N)*		N		0/1
	**R+180**		0.74 (0.69)^b^		
	*Returned to Baseline? (Y/N)*		N		0/1
	**R+360**		−0.15 (0.77)^b^		
	*Returned to Baseline? (Y/N)*		Y		1/1

**Notes.** data of outcome parameters related to skeletal system, reported as % change from baseline were extracted and displayed without any alterations for each reported recovery timepoint following a period of 6-degree-head-down-tilt bed rest. For each recovery timepoint the effect was categorize as “Returned to baseline” or “Not returned to baseline”. Returned to Baseline? = Y: Whenever the mean % change equals 0% or reverts from + to—/– to +; Returned to Baseline? = N: Whenever the mean % change remains + or –.All values are displayed as Mean % Change from Baseline (SD); BMC: bone mineral content; BMD: bone mineral density; Y: yes; N: no.

a Data extracted from figure using WebPlotDigitizer.

b Data presented as Mean (SEM); N.A.: not available.

### 3.5 Recovery of the ‘Muscular System’

**FIGURE 3:**
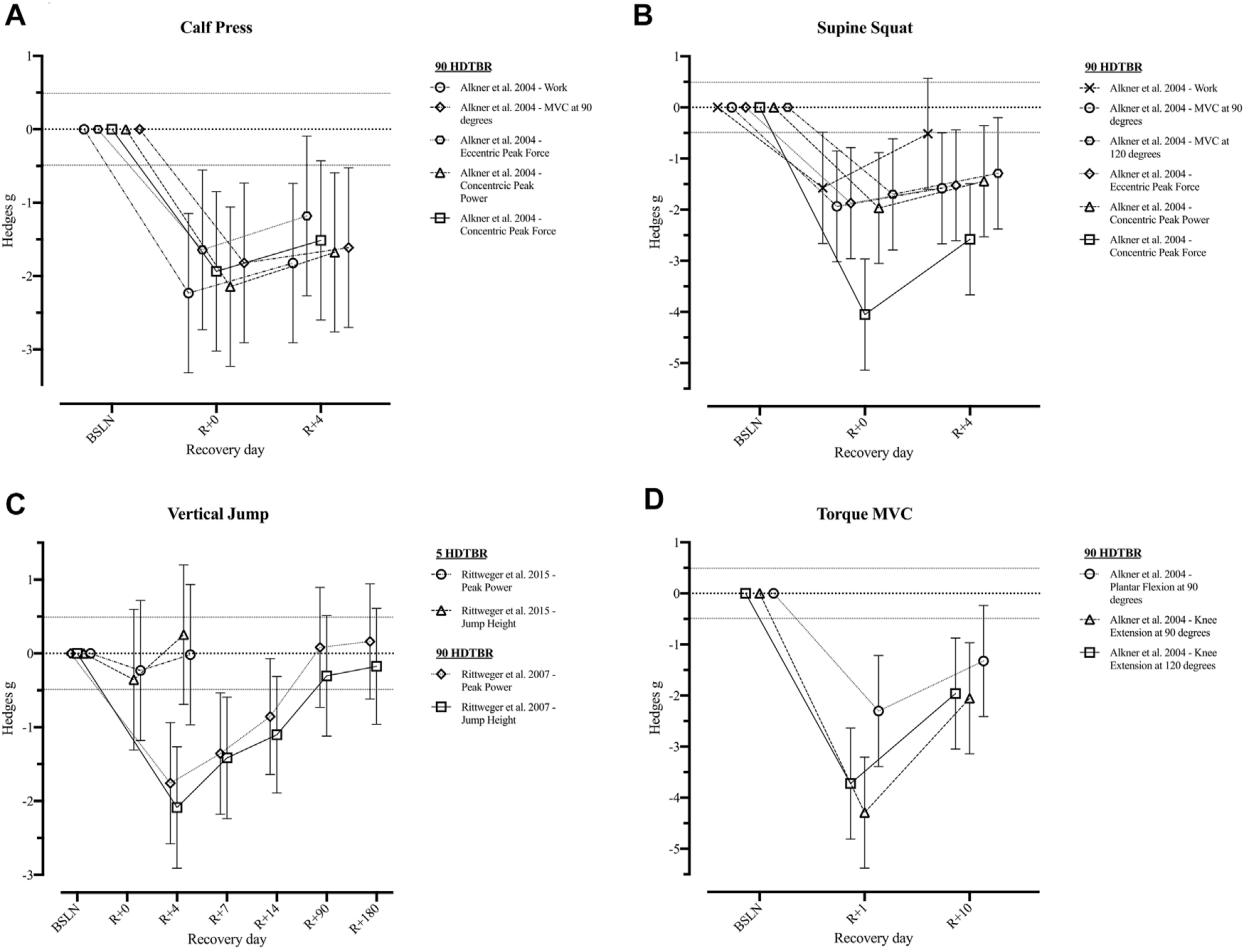
Visualisation of the recovery of outcomes related to the ‘*Muscular System*’ after a period of head-down-tilt bed rest, displayed as Hedges *g* with 95% Confidence Interval. **(A)** Recovery of outcomes related to the performance of a Calf Press **(B)** Recovery of outcomes related to the performance of a Supine Squat **(C)** Recovery of outcomes related to the performance of a Vertical Jump **(D)** Recovery of the Torque generated during performance of a Maximal Voluntary Contraction of the lower limb. To determine whether a particular outcome could be deemed as “recovered” during the recorded recovery period, the Westlake’s Confidence Interval Procedure (Seaman and Serlin, 1998) was used. This procedure tests for equivalence between two means using a confidence interval. To do so, Upper (0.49) and Lower (−0.49) equivalency bounds of interest were determined, corresponding to the limit of a small effect size. When combined with the 95% Confidence Interval of the Hedges *g*, three scenarios are possible: 1) *No evidence of recovery*: It cannot be concluded that the difference between means is trivial as the 95% Confidence Interval falls completely outside the set equivalency bounds; 2) *Weak evidence of recovery*: The results are inconclusive as the 95% Confidence Interval partially falls within the set equivalency bounds, thus including both trivial and non-trivial differences. The dotted lines at 0.49 and −0.49 represent the upper and lower equivalency bounds; 3) *Strong evidence of recovery:* There is practical equivalence as the 95% Confidence Interval falls completely within set equivalency bounds; 5 HDTBR: Included studies implementing a head-down-tilt bed rest period of 5 days (Rittweger et al., 2015); 90 HDTBR: Included studies implementing a head-down-tilt bed rest period of 90 days (Alkner and Tesch, 2004; Rittweger et al., 2007); R+…: Respective recovery day; MVC: Maximal Voluntary Contraction.

Three of the included studies (Rittweger et al., 2007, 2015; Alkner et al., 2016) reported a total of 16 operationally relevant outcomes related to the muscular system of which Hedges *g* effect sizes could be calculated (Figure 3 and *Supplementary Table S3*). Following a short HDTBR study of 5 days (Rittweger et al., 2015), jump height returned to baseline at R+4 (*g* = 0.25 [−0.69; 1.20]), while peak power during vertical jumping did not (*g* = −0.02 [−0.97; 0.93]). For the same parameters following a long duration HDTBR of 90 days (Rittweger et al., 2007), jump height did not return to baseline within the recorded recovery period of 180 days, while peak power did recover by R+90 (*g* = 0.08 [−0.73; 0.90]). Outcomes reported by Alkner et al. (2016) following a 90-days bed rest period did show improvement during the 10 days recorded recovery period, but did not fully recover.

One long duration HDTBR study (Belavý et al., 2017) reported the % change of lower limb muscle volumes which returned to baseline values by R+90, except for the recuts femoris muscle volume which only returned to baseline by R+360, following a 42-days bed rest period (Table 5). One medium (42 days; (Ferretti et al., 2001)) and one long (90 days; (Alkner et al., 2016)) duration study, although showing improvements in outcomes, did not report any of the outcomes to return to baseline within 4 (Alkner et al., 2016) to 48 days of recovery (Ferretti et al., 2001) (Table 5).

**TABLE 5:** % Change with SD of outcome parameters related to the *‘Muscular System*’.

	Recovery timepoint	Author			Returned to baseline? = Y
		Alkner et al. (2016)	Belavý et al. (2017)	Ferretti et al. (2001)	
		*90 days HDTBR*	*90 days HDTBR*	*42 days HDTBR*	
*Muscle Volume—Lateral Gastrocnemius*					
	**R+13**		−11.8 (11.6)		
	*Returned to Baseline? (Y/N)*		N		0/1
	**R+90**		2.6 (9.6)		
	*Returned to Baseline? (Y/N)*		Y		1/1
	**R+180**		1.1 (10.1)		
	*Returned to Baseline? (Y/N)*		Y		1/1
	**R+360**		3.0 (10.5)		
	*Returned to Baseline? (Y/N)*		Y		1/1
*Muscle Volume—Medial Gastrocnemius*					
	**R+13**		−7.6 (8.4)		
	*Returned to Baseline? (Y/N)*		N		0/1
	**R+90**		2.7 (6.4)		
	*Returned to Baseline? (Y/N)*		Y		1/1
	**R+180**		2.5 (4.8)		
	*Returned to Baseline? (Y/N)*		Y		1/1
	**R+360**		3.9 (6.0)		
	*Returned to Baseline? (Y/N)*		Y		1/1
*Muscle Volume—Soleus*					
	**R+13**		−5.5 (6.1)		
	*Returned to Baseline? (Y/N)*		N		0/1
	**R+90**		2.4 (4.6)		
	*Returned to Baseline? (Y/N)*		Y		1/1
	**R+180**		3.9 (3.2)		
	*Returned to Baseline? (Y/N)*		Y		1/1
	**R+360**		4.3 (5.0)		
	*Returned to Baseline? (Y/N)*		Y		1/1
*Muscle Volume—Vasti*					
	**R+13**		−10.1 (6.7)		
	*Returned to Baseline? (Y/N)*		N		0/1
	**R+90**		0.9 (7.5)		
	*Returned to Baseline? (Y/N)*		Y		1/1
	**R+180**		1.5 (6.8)		
	*Returned to Baseline? (Y/N)*		Y		1/1
	**R+360**		3.1 (8.5)		
	*Returned to Baseline? (Y/N)*		Y		1/1
*Muscle Volume—Rectus Femoris*					
	**R+13**		−4.2 (6.2)		
	*Returned to Baseline? (Y/N)*		N		0/1
	**R+90**		−0.8 (6.0)		
	*Returned to Baseline? (Y/N)*		N		0/1
	**R+180**		−0.1 (5.4)		
	*Returned to Baseline? (Y/N)*		N		0/1
	**R+360**		1.0 (6.9)		
	*Returned to Baseline? (Y/N)*		Y		1/1
*Muscle Volume—Biceps Femoris Long Head*					
	**R+13**		−10.5 (7)		
	*Returned to Baseline? (Y/N)*		N		0/1
	**R+90**		2.7 (6.9)		
	*Returned to Baseline? (Y/N)*		Y		1/1
	**R+180**		1.7 (4.9)		
	*Returned to Baseline? (Y/N)*		Y		1/1
	**R+360**		2.2 (6)		
	*Returned to Baseline? (Y/N)*		Y		1/1
*Muscle Volume—Biceps Femoris Short Head*					
	**R+13**		−0.2 (7.1)		
	*Returned to Baseline? (Y/N)*		N		0/1
	**R+90**		4.5 (8.5)		
	*Returned to Baseline? (Y/N)*		Y		1/1
	**R+180**		3 (7.8)		
	*Returned to Baseline? (Y/N)*		Y		1/1
	**R+360**		3.1 (8.4)		
	*Returned to Baseline? (Y/N)*		Y		1/1
*Muscle Volume—Semimembranosus*					
	**R+13**		−8.4 (6.5)		
	*Returned to Baseline? (Y/N)*		N		0/1
	**R+90**		2.4 (4.5)		
	*Returned to Baseline? (Y/N)*		Y		1/1
	**R+180**		2.7 (3.7)		
	*Returned to Baseline? (Y/N)*		Y		1/1
	**R+360**		4.3 (6)		
	*Returned to Baseline? (Y/N)*		Y		1/1
*Muscle Volume—Semitendinosus*					
	**R+13**		−3.6 (6)		
	*Returned to Baseline? (Y/N)*		N		0/1
	**R+90**		2.2 (5.8)		
	*Returned to Baseline? (Y/N)*		Y		1/1
	**R+180**		2.2 (6.4)		
	*Returned to Baseline? (Y/N)*		Y		1/1
	**R+360**		2 (7.6)		
	*Returned to Baseline? (Y/N)*		Y		1/1
*Muscle Volume—Popliteus*					
	**R+13**		-0.4 (8.2)		
	*Returned to Baseline? (Y/N)*		N		0/1
	**R+90**		2.4 (7.4)		
	*Returned to Baseline? (Y/N)*		Y		1/1
	**R+180**		−0.7 (6.2)		
	*Returned to Baseline? (Y/N)*		N		0/1
	**R+360**		1.8 (8.3)		
	*Returned to Baseline? (Y/N)*		Y		1/1
*Muscle Volume—Lower Gluteus Maximus*					
	**R+13**		−4.5 (6.4)		
	*Returned to Baseline? (Y/N)*		N		0/1
	**R+90**		1.6 (7.2)		
	*Returned to Baseline? (Y/N)*		Y		1/1
	**R+180**		0.8 (8.1)		
	*Returned to Baseline? (Y/N)*		Y		1/1
	**R+360**		5.5 (10.9)		
	*Returned to Baseline? (Y/N)*		Y		1/1
*Muscle Volume—Iliopsoas*					
	**R+13**		0.5 (9.5)		
	*Returned to Baseline? (Y/N)*		Y		1/1
	**R+90**		−1.3 (9)		
	*Returned to Baseline? (Y/N)*		N		0/1
	**R+180**		0.4 (10)		
	*Returned to Baseline? (Y/N)*		Y		1/1
	**R+360**		1.7 (9.7)		
	*Returned to Baseline? (Y/N)*		Y		1/1
*Maximal Voluntary Contraction—Quadriceps*					
	**R+0**	−45 (n.a.)			
	*Returned to Baseline? (Y/N)*	N			0/1
	**R+4**	−36 (n.a.)			
	*Returned to Baseline? (Y/N)*	N			0/1
*Maximal Absolute Muscle Power during Vertical Jump*					
	**R+2**			−23.7 (6.9)	
	*Returned to Baseline? (Y/N)*			N	0/1
	**R+6**			−20.9 (3.4)	
	*Returned to Baseline? (Y/N)*			N	0/1
	**R+48**			−3.8 (n.a.)	
	*Returned to Baseline? (Y/N)*			N	0/1
*Maximal Muscle Power normalized to body weight during Vertical Jump*					
	**R+2**			−22.7 (5.4)	
	*Returned to Baseline? (Y/N)*			N	0/1
	**R+6**			−20.2 (1.6)	
	*Returned to Baseline? (Y/N)*			N	0/1
	**R+48**			−4.7 (n.a.)	
	*Returned to Baseline? (Y/N)*			N	0/1
*Maximal Contraction Force from Vertical Jump*					
	**R+2**			−14.7 (5.5)	
	*Returned to Baseline? (Y/N)*			N	0/1
	**R+6**			−11.8 (5.2)	
	*Returned to Baseline? (Y/N)*			N	0/1

**Notes.** data of outcome parameters related to the muscular system, reported as % change from baseline were extracted and displayed without any alterations for each reported recovery timepoint following a period of 6-degree-head-down-tilt bed rest. For each recovery timepoint the effect was categorize as “Returned to baseline” or “Not returned to baseline”. Returned to Baseline? = Y: Whenever the mean % change equals 0% or reverts from + to—/– to +; Returned to Baseline? = N: Whenever the mean % change remains + or –.All values are displayed as Mean % Change from Baseline (SD); Y: yes; N: no. Data extracted from figure using WebPlotDigitizer. N.A.: not available.

### 3.6 Recovery of the ‘Cardiovascular System’

**FIGURE 4:**
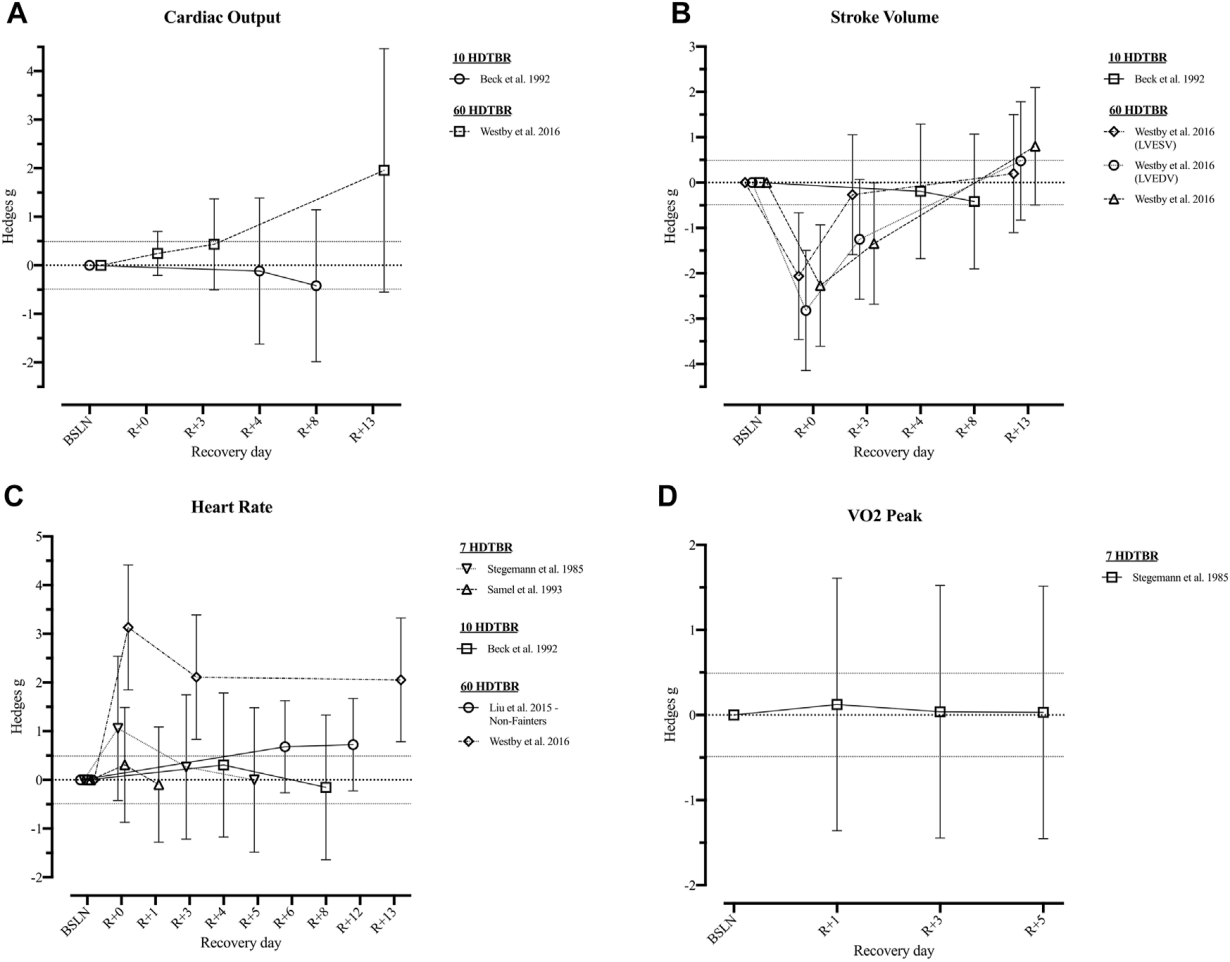
Visualisation of the recovery of outcomes related to the ‘*Cardiovascular System*’ (Part 1) after a period of head-down-tilt bed rest, displayed as Hedges *g* with 95% Confidence Interval. **(A)** Recovery of the Cardiac Output at rest **(B)** Recovery of outcomes related to the Stroke Volume at rest **(C)** Recovery of the resting Heart Rate **(D)** Recovery of the VO2 peak. To determine whether a particular outcome could be deemed as “recovered” during the recorded recovery period, the Westlake’s Confidence Interval Procedure (Seaman and Serlin, 1998) was used. This procedure tests for equivalence between two means using a confidence interval. To do so, Upper (0.49) and Lower (−0.49) equivalency bounds of interest were determined, corresponding to the limit of a small effect size. When combined with the 95% Confidence Interval of the Hedges *g*, three scenarios are possible: 1) *No evidence of recovery*: It cannot be concluded that the difference between means is trivial as the 95% Confidence Interval falls completely outside the set equivalency bounds; 2) *Weak evidence of recovery*: The results are inconclusive as the 95% Confidence Interval partially falls within the set equivalency bounds, thus including both trivial and non-trivial differences. The dotted lines at 0.49 and −0.49 represent the upper and lower equivalency bounds; 3) *Strong evidence of recovery:* There is practical equivalence as the 95% Confidence Interval falls completely within set equivalency bounds; 7 HDTBR: Included studies implementing a head-down-tilt bed rest period of 7 days (Stegemann et al., 1985; Samel et al., 1993); 10 HDTBR: Included studies implementing a head-down-tilt bed rest period of 10 days (Beck et al., 1992); 60 HDTBR: Included studies implementing a head-down-tilt bed rest period of 60 days (Liu et al., 2015; Westby et al., 2016); R+…: Respective recovery day; LVESV: Left Ventricular End Systolic Volume; LVEDV: Left Ventricular End Diastolic Volume.

**FIGURE 5:**
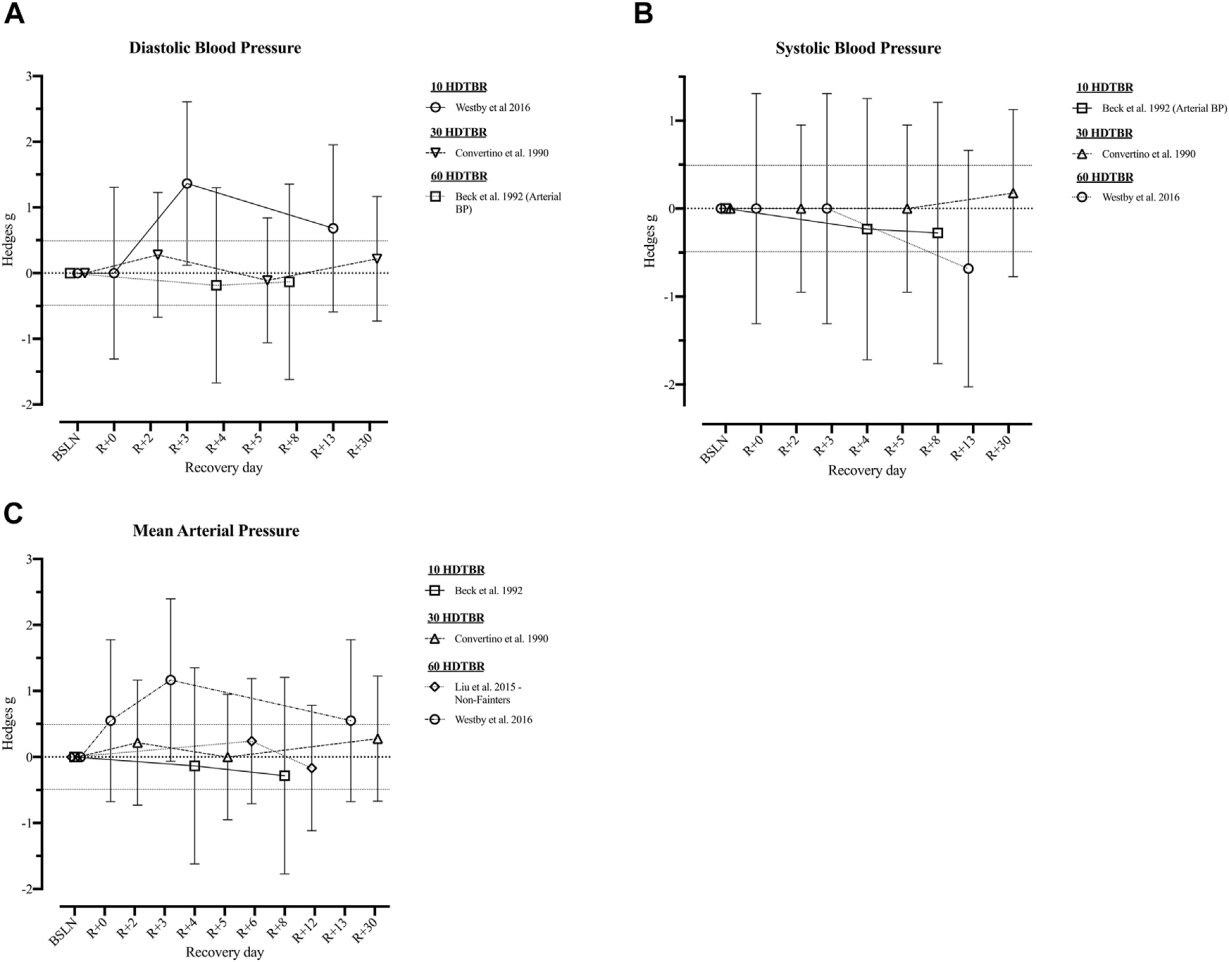
Visualisation of the recovery of outcomes related to the ‘*Cardiovascular System*’ (Part 2) after a period of head-down-tilt bed rest, displayed as Hedges *g* with 95% Confidence Interval. **(A)** Recovery of the Diastolic Blood Pressure **(B)** Recovery of the Systolic Blood Pressure **(C)** Recovery of Mean Arterial Pressure. To determine whether a particular outcome could be deemed as “recovered” during the recorded recovery period, the Westlake’s Confidence Interval Procedure (Seaman and Serlin, 1998) was used. This procedure tests for equivalence between two means using a confidence interval. To do so, Upper (0.49) and Lower (−0.49) equivalency bounds of interest were determined, corresponding to the limit of a small effect size. When combined with the 95% Confidence Interval of the Hedges *g*, three scenarios are possible: 1) *No evidence of recovery*: It cannot be concluded that the difference between means is trivial as the 95% Confidence Interval falls completely outside the set equivalency bounds; 2) *Weak evidence of recovery*: The results are inconclusive as the 95% Confidence Interval partially falls within the set equivalency bounds, thus including both trivial and non-trivial differences. The dotted lines at 0.49 and −0.49 represent the upper and lower equivalency bounds; 3) *Strong evidence of recovery:* There is practical equivalence as the 95% Confidence Interval falls completely within set equivalency bounds; 10 HDTBR: Included studies implementing a head-down-tilt bed rest period of 10 days (Westby et al., 2016); 30 HDTBR: Included studies implementing a head-down-tilt bed rest period of 30 days (Convertino et al., 1990); 60 HDTBR: Included studies implementing a head-down-tilt bed rest period of 60 days (Beck et al., 1992; Liu et al., 2015; Westby et al., 2016); R+…: Respective recovery day; Arterial BP**:** Arterial systolic and diastolic Blood Pressure.

Following short duration HDTBR, cardiac output, stroke volume, systolic and diastolic blood pressure, and mean arterial pressure remained decreased at R+8 following 10 days bed rest (Beck et al., 1992) Resting heart rate on the other hand returned to baseline between R+1 (Samel et al. (1993); 7 days HDTBR) and R+8 (Beck et al. (1992); 10 days HDTBR). Following a 7-days HDTBR (Stegemann et al., 1985), VO2 peak measured while using a bicycle ergometer revealed an initial increase at R+1 (*g* = 0.12 [−1.36; 1.61]) but decreased during the following days (R+5: *g* = 0.03 [−1.45; 1.51]). Details on recovery following medium duration HDTBR were limited to the diastolic and systolic blood pressure and mean arterial pressure, as reported by Convertino et al. (1990), which elevated throughout the recovery period (R+2—R+30). Recovery of cardiovascular outcomes after long duration (60-days) HDTBR were reported by Westby et al. (2016) and Liu et al. (2015). Results of Westby et al. (2016) indicated cardiac output was elevated on R+0 (*g* = 0.25 [−0.21; 0.70]) and increased during the following days (R+13: *g* = 1.96 [−0.55; 4.46]). The same is noted for stroke volume and the left ventricular end systolic/diastolic volume, although at R+0 a reduction is noted (*g* = −2.27 [−3.61; 0.93]; −2.06 [−3.46; −0.66] and −2.82 [−4.15; −1.49] respectively) baseline values are surpassed at R+13 (*g* = 0.80 [−0.49; 2.10]; 0.20 [−1.10; 1.50] and 0.48 [−0.83; 1.78] respectively). Results on the recovery of heart rate at rest are contradictory as Westby et al. (2016) reported a decrease during the 13-days recovery period, while Liu et al. (2015) reported an increase during the 12-days recovery period, the same trend could be noted for the recovery of the mean arterial pressure. Hedges *g* values of the included outcomes are presented in Figures 4, 5 (*Supplementary Table S4*).

Reportings on the percentage change from baseline were limited to heart rate, systolic and diastolic blood pressure and mean arterial pressure (Table 6). Following a 10-days HDTBR period (Schulz et al., 1992), heart rate showed an increase from R+0 to R+15, remained stable during the first 14 days post 60-days HDTBR (Kramer et al., 2017), and did not change within the initial 3 days of recovery following 120 days of HDTBR (Linnarsson et al., 2006). For systolic blood pressures, a reduction was noted from R+1 to R+14 after 60 days HDTBR (Kramer et al., 2017), while staying stable but elevated during the initial 14 days of recovery following 120 days of bed rest (Linnarsson et al., 2006), which was also the case for diastolic blood pressure (Kramer et al., 2017). Mean arterial pressure showed a slight decrease during the initial 4 days of recovery following 10 days of HDTBR (Schulz et al., 1992), while remaining elevated up to R+14 after 120 days of bed rest (Linnarsson et al., 2006).

**TABLE 6:** % Change with SD of outcome parameters related to the *‘Cardiovascular System*’.

	Recovery timepoint	Author			Returned to baseline? = Y
		Kramer et al. (2017)	Linnarsson et al. (2006)	Schulz et al. (1992)	
		*60 days HDTBR*	*120 days HDTBR*	*10 days HDTBR*	
*Heart Rate*					
	**R+0**	13.05 (13.05) ^a^	12.73 (n.a)^a^	2 (9)	
	*Returned to Baseline? (Y/N)*	N	N	N	0/3
	**R+1**	32.47 (14.98) ^a^			
	*Returned to Baseline? (Y/N)*	N			0/1
	**R+2**	24.18 (15.65) ^a^			
	*Returned to Baseline? (Y/N)*	N			0/1
	**R+3**	22.15 (13.17) ^a^	12.73 (n.a.)^a^		
	*Returned to Baseline? (Y/N)*	N	N		0/2
	**R+4**	14.26 (12.32) ^a^		-1 (10)	
	*Returned to Baseline? (Y/N)*	N		Y	1/2
	**R+5**	12.43 (8.56) ^a^			
	*Returned to Baseline? (Y/N)*	N			0/1
	**R+6**	8.85 (9.93) ^a^			
	*Returned to Baseline? (Y/N)*	N			0/1
	**R+7**	10.71 (7.27) ^a^			
	*Returned to Baseline? (Y/N)*	N			0/1
	**R+8**	13.86 (10.51) ^a^			
	*Returned to Baseline? (Y/N)*	N			0/1
	**R+9**	6.68 (8.56) ^a^			
	*Returned to Baseline? (Y/N)*	N			0/1
	**R+10**	13.01 (12.50) ^a^			
	*Returned to Baseline? (Y/N)*	N			0/1
	**R+11**	8.32 (8.99) ^a^			
	*Returned to Baseline? (Y/N)*	N			0/1
	**R+12**	10.13 (15.96) ^a^			
	*Returned to Baseline? (Y/N)*	N			0/1
	**R+13**	11.24 (10.68) ^a^			
	*Returned to Baseline? (Y/N)*	N			0/1
	**R+14**	12.04 (8.56) ^a^			
	*Returned to Baseline? (Y/N)*	N			0/1
	**R+15**		9.17 (n.a)^a^		
	*Returned to Baseline? (Y/N)*		N		0/1
*Systolic Blood Pressure*					
	**R+0**	1.65 (3.15) ^a^	10.94 (n.a.) ^a^		
	*Returned to Baseline? (Y/N)*	N	N		0/1
	**R+1**	−2.31 (5.39) ^a^			
	*Returned to Baseline? (Y/N)*	Y			1/1
	**R+2**	−4.24 (5.45) ^a^			
	*Returned to Baseline? (Y/N)*	Y			1/1
	**R+3**	−3.16 (6.81) ^a^	10.41 (n.a.) ^a^		
	*Returned to Baseline? (Y/N)*	Y	N		1/2
	**R+4**	−2.88 (6.20) ^a^			
	*Returned to Baseline? (Y/N)*	Y			1/1
	**R+5**	−2.65 (6.77) ^a^			
	*Returned to Baseline? (Y/N)*	Y			1/1
	**R+6**	−5.21 (7.38) ^a^			
	*Returned to Baseline? (Y/N)*	Y			1/1
	**R+7**	−8.27 (5.61) ^a^			
	*Returned to Baseline? (Y/N)*	Y			1/1
	**R+8**	−4.30 (4.39) ^a^			
	*Returned to Baseline? (Y/N)*	Y			1/1
	**R+9**	−8.28 (4.06) ^a^			
	*Returned to Baseline? (Y/N)*	Y			1/1
	**R+10**	−7.23 (3.45) ^a^			
	*Returned to Baseline? (Y/N)*	Y			1/1
	**R+11**	−5.87 (5.00) ^a^			
	*Returned to Baseline? (Y/N)*	Y			1/1
	**R+12**	−5.03 (7.40) ^a^			
	*Returned to Baseline? (Y/N)*	Y			1/1
	**R+13**	−5.05 (4.23) ^a^			
	*Returned to Baseline? (Y/N)*	Y			1/1
	**R+14**	−4.56 (6.24) ^a^	10.32 (n.a.) ^a^		
	*Returned to Baseline? (Y/N)*	Y	N		1/2
*Diastolic Blood Pressure*					
	**R+0**	6.48 (4.32) ^a^			
	*Returned to Baseline? (Y/N)*	N			0/1
	**R+1**	2.74 (6.58) ^a^			
	*Returned to Baseline? (Y/N)*	N			0/1
	**R+2**	−3.94 (6.58) ^a^			
	*Returned to Baseline? (Y/N)*	Y			1/1
	**R+3**	−4.45 (12.03) ^a^			
	*Returned to Baseline? (Y/N)*	Y			1/1
	**R+4**	−5.42 (9.16) ^a^			
	*Returned to Baseline? (Y/N)*	Y			1/1
	**R+5**	−2.90 (7.90) ^a^			
	*Returned to Baseline? (Y/N)*	Y			1/1
	**R+6**	−7.35 (11.16) ^a^			
	*Returned to Baseline? (Y/N)*	Y			1/1
	**R+7**	−11.61 (8.90) ^a^			
	*Returned to Baseline? (Y/N)*	Y			1/1
	**R+8**	−9.55 (6.90) ^a^			
	*Returned to Baseline? (Y/N)*	Y			1/1
	**R+9**	−12.42 (4.84) ^a^			
	*Returned to Baseline? (Y/N)*	Y			1/1
	**R+10**	−9.32 (4.84) ^a^			
	*Returned to Baseline? (Y/N)*	Y			1/1
	**R+11**	−7.07 (5.84) ^a^			
	*Returned to Baseline? (Y/N)*	Y			1/1
	**R+12**	−5.61 (9.55) ^a^			
	*Returned to Baseline? (Y/N)*	Y			1/1
	**R+13**	−7 (6.29) ^a^			
	*Returned to Baseline? (Y/N)*	Y			1/1
	**R+14**	−4.65 (6.77) ^a^			
	*Returned to Baseline? (Y/N)*	Y			1/1
*Mean Arterial Pressure*					
	**R+0**		4.92 (n.a.) ^a^	-6 (5)	
	*Returned to Baseline? (Y/N)*		N	N	0/2
	**R+3**		4.52 (n.a.) ^a^		
	*Returned to Baseline? (Y/N)*		N		0/1
	**R+4**			-4 (5)	
	*Returned to Baseline? (Y/N)*			N	0/1
	**R+15**		4.92 (n.a.) ^a^		
	*Returned to Baseline? (Y/N)*		N		0/1

**Notes.** data of outcome parameters related to the cardiovascular system, reported as % change from baseline were extracted and displayed without any alterations for each reported recovery timepoint following a period of 6-degree-head-down-tilt bed rest. For each recovery timepoint the effect was categorize as “Returned to baseline” or “Not returned to baseline”. Returned to Baseline? = Y: Whenever the mean % change equals 0% or reverts from + to—/– to +; Returned to Baseline? = N: Whenever the mean % change remains + or –.All values are displayed as Mean % Change from Baseline (SD); Y: yes; N: no.

a Data extracted from figure using WebPlotDigitizer.

## 4 Discussion

As the passive control groups of HDTBR well represented the concept of exercise holiday, we set out to explore the recovery dynamics of this group rather than elaborating on ‘adequate’ or ‘inadequate’ exercise-countermeasures of the intervention group. By doing so, the potential advantages of exercise holidays in accordance with the needs of and shifts in future crewed space missions could be highlighted, to be included and explored upon in future evidence-based countermeasure programmes. Main findings on the post-HDTBR recovery dynamics of the passive control group include: 1) anthropometric outcomes show steady improvements, with a possible return to baseline between R+3 and R+90; 2) recovery of BMC and BMD of the lower limbs reveals a continued decrease up to R+14, followed by steady improvements but failing to fully recover by R+360; 3) lower limb muscle volumes show a consistent recovery by R+90; and 4) independent of HDBTR campaign duration, cardiovascular outcomes showed trends of normalization within the initial 14 days of recovery.

As future LEO and exploration missions will differ in duration, for the sake of clarity, the current literature will primarily be discussed considering the upcoming Artemis and Lunar Gateway missions that are slated to take between 30 and 90 days (Gerstenmaier and Crusan, 2018; NASA, 2021). Such mission durations are consistent with the HDTBR durations used in almost all of the included literature: 30 days (Convertino et al., 1990), 42 days (Ferretti et al., 2001), 60 days (Belavý et al., 2011a; Beller et al., 2011; Liu et al., 2015; Westby et al., 2016; Kramer et al., 2017), and 90 days (Alkner and Tesch, 2004; Rittweger et al., 2007; Rittweger and Felsenberg, 2009; Alkner et al., 2016; Belavý et al., 2017). The remaining six HDTBR studies were of shorter duration: 5 days (Rittweger et al., 2015), 7 days (Stegemann et al., 1985; Samel et al., 1993), 10 days (Beck et al., 1992; Schulz et al., 1992), except one which was 120 days (Linnarsson et al., 2006).

### 4.1 Anthropometric Outcomes

In space, loss of body mass appears to be highly variable, but the average rate has been estimated to be around 2.4% per 100 days spent in space (Matsumoto et al., 2011). However, this must be contextualised by the fact that astronauts are all performing extensive countermeasures (Petersen et al., 2016). In contrast, current HDTBR data without exercise countermeasures demonstrates reduced body mass, during and shortly after long duration HDTBR (Hedges *g* = [−0.43; −0.25], Figure 2 *,* (Rittweger et al., 2007; Westby et al., 2016; Kramer et al., 2017)). Body mass decrements may be precipitated by changes in blood volume (Tavassoli, 1982; Kunz et al., 2017), muscle atrophy (LeBlanc et al., 2000; Alkner and Tesch, 2004; Winnard et al., 2019) and/or bone demineralization (LeBlanc et al., 2000; Rittweger et al., 2005; Belavý et al., 2011b). A potentially important driver for body mass loss may be negative energy balance due to the mismatch between energy intake and energy expenditure (Stein, 2000; Laurens et al., 2019). Data presented by Stein (2000) suggests a moderate positive relationship between the total energy expenditure and loss of body mass during spaceflight. Thus, the increased energy expenditure associated with exercise countermeasures appears not to be accompanied by increased energy intake, resulting in a negative energy balance. In fact, a negative nitrogen balance—suggesting loss of muscle mass—was also reported in-flight—despite performing exercise countermeasures (Stein, 2000; Stein, 2013). However, interestingly during the first 2 weeks of the Space Life Sciences (SLS) 1 and 2 Shuttle missions (Stein et al., 1996) where no exercise countermeasures were performed, energy and nitrogen balance were stable, suggesting a muscle mass preservation (Stein, 2000).

In contrast, following 90-days HDTBR, Rittweger et al. (2007) reported that body mass loss was still apparent after 14 days of recovery (Hedges *g* = −0.25). Similarly, following 60-days HDTBR, Kramer et al. (2017) reported approximately 5% body mass reductions, mostly attributed to lean body mass loss which also did not recover within 14 days post-HDTBR. This body mass loss disparity may be due to the energy intake reported by Kramer et al. (2017) being calculated based on the resting metabolic rate, instead of the actual 24-h energy expenditure (Piaggi et al., 2015; Laurens et al., 2019). In contrast, Westby et al. (2016) adjusted daily caloric intake so that body mass was maintained within 3% of that on the third day of HDTBR. This resulted in the body mass returning to baseline after the third day of recovery (Hedges *g* = 0.00, Figure 2). Thus, depending on HDTBR duration and dietary intake, body mass recovery may occur from 3 days (Westby et al., 2016) up to 3 months, or longer (Rittweger et al., 2007). Yet, based on data of 246 different astronauts over 514 mission, 62% failed to regain all of the lost body mass at a time-interval of R+[91–396] days postflight (Matsumoto et al., 2011), unfortunately, relative changes in lean and adipose body mass are unknown. Sustained reductions in body mass could however contribute to a significant risk of an adverse effects such as reduced stamina or increased risk of muscle injuries (Matsumoto et al., 2011). Especially if loss of lean body mass is evident and results in an operationally meaningful loss of muscle strength (i.e., considerable and in a specific muscle group), thus possibly leading to a crewmember not being able to perform an operational task that they previously could.

### 4.2 The Skeletal System

In space, the average rate of bone loss has been estimated to be between 0.5% (Stavnichuk et al., 2020) and 1.5% (Lang et al., 2004) per month in the lower limbs, despite in-flight exercise countermeasures (Smith et al., 2012). Thus, the rate of bone loss would presumably be even greater if no countermeasures were being performed. During 60-days HDTBR, Beller et al. (2011) reported tibial bone mineral density loss ranging between 1.1 and 2.0% per month, while for the hip BMD decreased by between 1.5 and 2.0% per month, potentially increasing the risk of fractures. Although BMD loss appears to be slightly greater during HDTBR—with no exercise countermeasures—fortunately it remains far below that observed in spinal cord injury patients. In this cohort, a rapid linear decline of lower extremity BMD results in a loss of ∼27% in the first three to 4 months after injury and reaching a plateau at ∼37% after 16 months (Biering-Sorensen et al., 1990; Garland et al., 1992), thus substantially increasing the risk of bone fractures (Gernand, 2004).

However, the limited data presented in the current study suggests that bone recovery is slow, potentially taking up to 3 to 4 times that of the unloading period (Gernand, 2004; Orwoll et al., 2013; Stavnichuk et al., 2020). In fact, long duration HDTBR data on lower limb bone mineral content (Rittweger and Felsenberg, 2009; Belavý et al., 2011a) and density (Beller et al., 2011) suggests that the loss continues up to a period of 14 days after HDTBR is concluded (Table 4) due to the inertia in bone remodelling regulation. Similarly, bone accrual appears to be evident only after approximately 1 week of reconditioning (Armbrecht et al., 2010). Furthermore, some residual BMD loss appears to persist, which may increase long-term fracture risk. Decrements of BMD of the tibia (−1.81 ± 0.82%) and the hip (−0.52 ± 0.65%) were still present at R+360 after a 60-days HDTBR (Beller et al., 2011). Similarly, loss of BMD postflight was still present 6 months after long-duration spaceflight (Vico et al., 2000), and was even persistent after 5 years in nine Skylab crew members (Tilton et al., 1980). Moreover, Sibonga et al. (2007) determined the ‘50% recovery time’ based on data of 46 long-duration crew members assigned to Mir or ISS missions. This 50% recovery time represents the number of days after landing, needed to restore half of the lost BMD and ranged between 97 days for the Pelvis and 255 days for the Trochanter. Whilst small increases in bone fracture risk may be acceptable when returning to Earth, this could be critical when landing and performing extravehicular activities (EVAs) on the Lunar surface in the absence of medical support (Horneck et al., 2003). Therefore, limits of acceptable losses of BMD—within a spaceflight context—should be defined, as for example has been done for osteopenia (BMD T-score: −2.5 < T-score < −1.0) or osteoporosis (BMD T-score < −2.5) (Woolf and Pfleger, 2003). Determining how close a person gets to a significant increase in risk of low trauma fractures during a period of exercise-free bed rest—and the recovery thereof—is key in determining whether this limit of acceptable bone loss is equal to a pathological threshold (i.e., osteopenia or osteoporosis), or whether an acceptable operational threshold is closer to normal.

### 4.3 The Muscular System

In space, as with the skeletal system, the muscles most affected are those with a prime ‘anti-gravitational’ function such as those in the trunk and lower limbs (Stein, 2013; Winnard et al., 2019). Based on the data presented in the review of Winnard et al. (2019) moderate effects (Hedges *g* ≥ 0.6) occur within seven to 14 days of HDTBR, while large effects (Hedges *g* ≥ 1.2) occur after 28–35 days. Muscle mass is critical to a crewmember’s strength and endurance (Gernand, 2004; Winnard et al., 2019). In general, large effects (i.e., reduction) of muscle volume and cross-sectional area were only noted after 28 days of HDTBR, whereas decrements of muscle thickness, maximal torque, and strength after 35 days, whilst large peak power effects were apparent after 56 days (Winnard et al., 2019). Such decrements could impede mission critical tasks such as EVAs or landing operations.

Information on the recovery of maximal voluntary contractions, peak forces, or the work performed during a supine squat or calf press was only reported up until the fourth day of recovery after a 90-day period of HDTBR (Alkner et al., 2016). Although improvements in all related outcomes were noted within 4 days, none returned to baseline (Hedges *g* = [−2.58; −0.51], Figure 3). Based on the information provided by Rittweger et al. (2007) on the peak power generated during jumping after 90-days HDTBR, it could be suggested that muscle outcomes recover within 90 days of recovery. Similarly, data reported by Belavý et al. (2017) after a 90-days HDTBR period indicates that lower limb muscle volume returns to baseline between day 13 and 90 of recovery (Table 5). This data concurs with findings of crewmembers returning from a long-duration spaceflight. Restoration of muscle mass and strength of crewmembers during the post-flight rehabilitation period seems to occur at the same rate, or even at a faster rate, of the initial atrophy (Leblanc et al., 1990; Tesch et al., 2005; Petersen et al., 2017). Thus, definition of the imposition of an exercise holiday should consider the high degree of inter-individual variability expressed in muscle outcomes (Gernand, 2004; Stein, 2013; Winnard et al., 2019). Consideration of relative effects should be made as ‘stronger’ crewmembers may be able to retain operational functionality whilst experiencing greater absolute and relative decrements of their pre-flight muscle mass and strength compared to those with lower pre-flight levels. Definition of ‘minimal’ strength requirements for spaceflight are critical to inform the implementation of any form of exercise holiday but have yet to be determined (Winnard et al., 2019).

### 4.4 The Cardiovascular System

Lastly, in space, cardiovascular system outcomes are significantly modulated to adapt to microgravity that negates hydrostatic gradients (Thornton et al., 1987). These changes in blood volume (Beck et al., 1992; Westby et al., 2016; Gallo et al., 2020), cardiac mass (Levine et al., 1997; Westby et al., 2016; Gallo et al., 2020), and aerobic capacity (Levine et al., 1996; Gallo et al., 2020) can be detrimental when returning to Earth or another celestial body. In fact, one of the most common consequences after long duration spaceflight is orthostatic intolerance (Hargens and Richardson, 2009; Liu et al., 2015) which could be critical during landing (Buckey, 2006). Orthostatic intolerance, with associated hypotension and presyncope, usually takes between 3 days (Waters et al., 2002) and 2 weeks (Vasilyeva and Bogomolov, 1991; Cooke et al., 2000) to recover following long-duration spaceflight. However, Fu et al. (2019) reported that contrary to tilt-table testing and still-standing, after 6 months in space, none of the 12 tested astronauts experienced orthostatic intolerance or hypotension during activities of daily living during the initial 24 h on Earth. While after 60-days HDTBR three out of 14 subjects were reported as ‘fainters’ during a head-up tilt test immediately after bed rest (Liu et al., 2015). Thus, non-exercise countermeasures such as volume resuscitation through water intake (Fu et al., 2019), or repeated exposure to Lower Body Negative Pressure (LBNP) used to mitigate spaceflight-associated neuro-ocular syndrome (SANS) (Harris et al., 2020) may be helpful to reduce cardiovascular deconditioning including orthostatic intolerance (Watenpaugh et al., 2007; Harris et al., 2020) which would support exercise holiday feasibility.

Additionally, some evidence suggests that aerobic capacity decrements are rapid during the first 30 days of spaceflight, after which (with exercise countermeasures) adaptations appear to plateau (Gernand, 2004; Gallo et al., 2020). Aerobic capacity losses may be an issue for EVAs with even moderate reductions potentially limiting a crewmembers’ ability to perform Lunar surface operations (Moore et al., 2014). However, with exercise there is increasing evidence to suggest that aerobic capacity recovers, at least in part, in-flight (Gernand, 2004; Moore et al., 2014; Gallo et al., 2020) and with complete recovery within 30 days postflight (Moore et al., 2014). Similarly, a recovery period of 14–30 days is reported in medium duration (20–42 days) HDTBR participants exposed to exercise countermeasures (Convertino et al., 1985; Sundblad et al., 2000). Also following 60-days HDTBR without exercise countermeasures, cardiac mass and function recovered within 14 days (Table 6) when participants were subjected to a progressive reconditioning programme (Westby et al., 2016). In contrast, Beck et al. (1992) observed—after a 10-days HDTBR—decrements in cardiac output, stroke volume and blood pressure which remained lower than baseline at recovery day 8 when recovery was not supervised. Yet, reports of the recovery of peak oxygen uptake, a key metric of cardiovascular fitness, is currently lacking as only Stegemann et al. (1985) reported values after a 7-days HDTBR without exercise, which remained unchanged (Hedges *g* = [0.03; 0.12]). Based on post-spaceflight data (Perhonen et al., 2001; Trappe et al., 2006), cardiorespiratory responses, heart rate, stroke volume and left ventricular mass are expected to recover during the post-flight rehabilitation phase (Payne et al., 2007).

Importantly, recovery of cardiovascular outcomes could be enhanced when combined with non-exercise countermeasures such as Lower Body Negative Pressure (Harris et al., 2020) and/or fluid volume supplementation (Waters et al., 2005) which are already being implemented in current spaceflight operations (Fu et al., 2019) to minimize the risk of orthostatic intolerance if gravitational loading is to be re-imposed. Additionally, cardiovascular rehabilitation following a protracted period of exercise-free HDTBR may be rapid if the recovery period includes an individualized reconditioning programme (Westby et al., 2016). Future HDTBR studies should therefore aim to investigate the effects of standardized reconditioning programmes during and/or following long-duration HDTBR to increase the evidence base towards implementation of exercise holidays within a spaceflight context.

### 4.5 Reported Reconditioning Approach After HDTBR Vs Post-spaceflight

In addition to re-exposure to a nominal 1 g loading upon termination of bed rest in HDTBR-participants, and after returning to Earth’s gravity in crewmembers, they are subjected to a period of physical reconditioning. Yet, while the reconditioning of crewmembers following spaceflight is well-described (Petersen et al., 2017), most HDTBR studies failed to report any specifics on reconditioning or rehabilitation protocols used during the recovery period. Thirteen out of the 18 included studies only reported the duration of the recovery period without any additional details. One 30-days HDTBR-study reported a 5 day recovery period within the bed rest facility, followed by 25 days (R+6 to R+30) of uncontrolled recovery (Convertino et al., 1990). Kramer et al. (2017) reported that participants were restricted to free movement within the ward during the 15 days recovery period. In Rittweger et al. (2007) and Rittweger and Felsenberg (2009) participants were residing within the facility for 14 days after reambulation during which nutrition was controlled. Only Westby et al. (2016) reported a supervised and progressive reconditioning programme for 10 days, starting at R+4, which included a 1-h supervised ambulation and exercise programme. Throughout the reconditioning period, the intensity, duration, and complexity of the exercises were increased according to the tolerance of the subject with regards to foot tenderness and ankle and knee pain due to the prolonged bed rest. Such an approach demonstrated the recovery of the cardiac mass and function within 2 weeks following a 60-days HDTBR campaign.

The reconditioning approach of Westby et al. (2016) is similar to the highly individualized reconditioning programme used for each ESA crewmember as described by Petersen et al. (2017). In short, each crewmember is supported by a reconditioning team, including an experienced exercise specialist/sport scientist and a physiotherapist. The supervised post-flight reconditioning programme integrates various physiotherapeutic methods and elements from sports and exercise science, resulting in a comprehensive and highly individualized reconditioning programme lasting 21 days. Exercise sessions have a focus on promoting functionality, efficacy, safety, and adequate intensity to optimise neuromusculoskeletal and cardiovascular responses. As large inter-individual variations in postflight condition occur between astronauts, the daily 2-h sessions are adapted to the individual with regards to complexity and intensity. However, the aim for all crewmembers is to be able to perform near, or at the same pre-flight intensity by the end of the 21-days reconditioning programme. Such an intensive post-flight rehabilitation programme is sufficient to make a full recovery of most, but not all aspect of function. Therefore, this is then followed by unsupervised training using an individualised exercise programme aimed at improving, and maintaining, health and fitness over the following months by supporting the neuro-musculoskeletal regeneration process.

This lack of general reporting—or even implementation of—standardized methods or exercise prescriptions during the recovery after HDTBR is an important shortcoming, thus having a profound effect on the ability to compare results across bed rest studies, and to compare the recovery dynamics after prolonged HDTBR with those after actual spaceflight.

### 4.6 Limitations of the Included Studies

Firstly, although HDTBR is the most robust ground-based analogue to study the effects of prolonged gravitational unloading (Hargens and Vico, 2016), potential confounding factors related to Earth-based analogues need to be taken into account: the inability to completely abolish gravitational stress, and the absence of exposure to space radiation. Although similarities are observed between HDTBR and actual spaceflight, reported changes may appear more rapidly and be more severe during spaceflight as compared to bed rest. Yet, HDTBR is still considered to be a valid analogue despite these limitations (Pavy-Le Traon et al., 2007).

The 18 included studies reported a total of 49 relevant outcome variables across the domains of interest with heterogeneous measurement time points—particularly evident during the recovery periods. This diversity was compounded by a general paucity of data. In addition, inconsistent reporting of mean raw values with standard deviations limited the ability to calculate effect sizes. Thus, effect sizes were only calculated for 27 of the 49 included outcome variables. Even where sufficient information was provided, typically reported sample sizes were low–meaning that caution should be exercised when interpreting this data (Lakens, 2013).

Additionally, vote counting based on the direction of effects was also severely limited due to the inconsistent and heterogenous reporting of outcome measures, with only 4/32 outcome variables reported as percentage change being reported at least twice, thus seriously restricting the generalisability of results. Moreover, differences in baseline reference conditions, especially in cardiovascular outcome variables (e.g., measured while upright/sitting/supine), impairs comparison between studies (Norsk, 2020).

Lastly, significant shortcomings in—the reporting of—the used methodology were indicated by the poor results of quality appraisal of the bed rest methods of included studies (Winnard and Nasser, 2017), thus limiting their comparability.

### 4.7 Filling in the Gaps—Recommendations for Future Research

**FIGURE 6:**
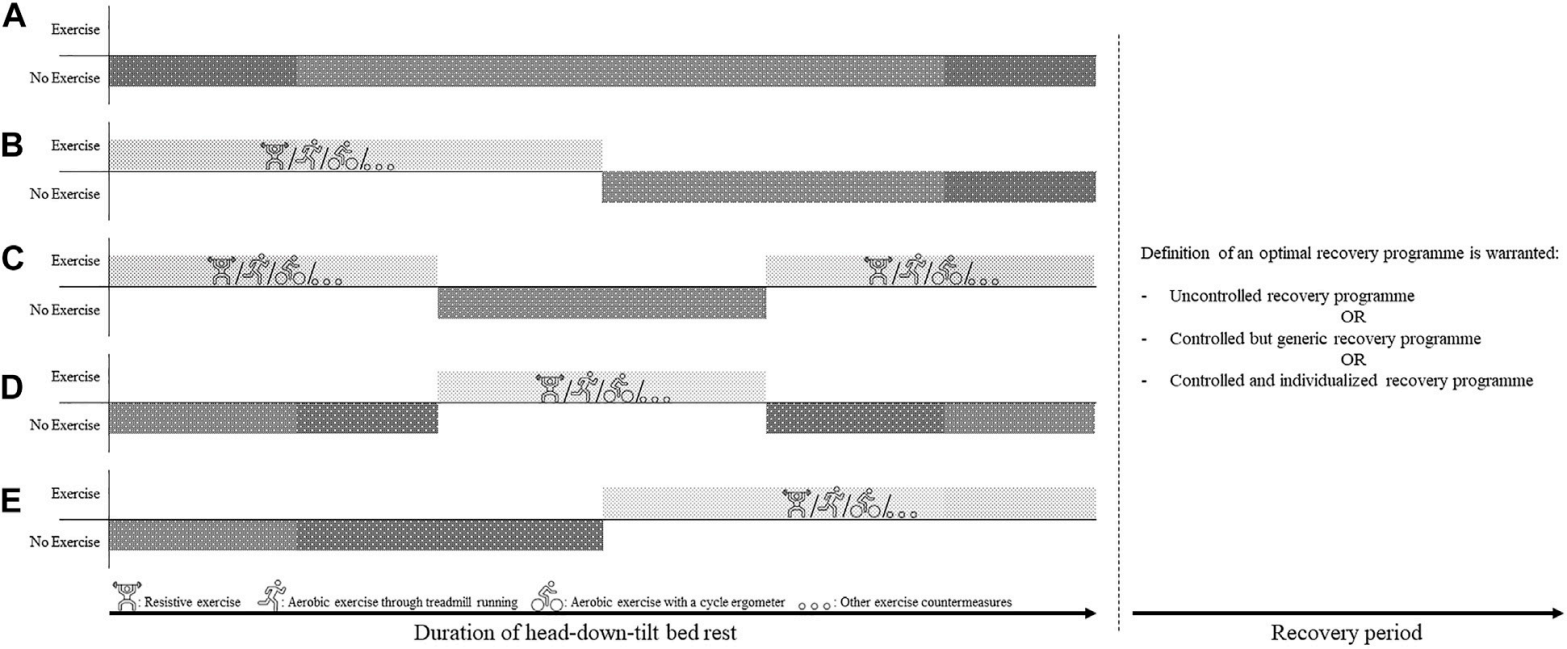
Illustration of possible implementations of Exercise Holidays and possible recovery programme modalities for future head-down-tilt bed rest campaigns. **(A) No Exercise:** The generic ‘no exercise’ control group wherein participants do not perform any exercise countermeasures through the duration of the bed rest campaign **(B) Exercise First:** Participants start the bed rest campaign with an exercise countermeasure programme, followed by a period of no exercise until the end of the campaign **(C) Exercise—Rest—Exercise:** Participants start and end the bed rest campaign with a period of exercise countermeasures, separated by a period of exercise holidays **(D) Rest—Exercise—Rest:** Participants start and end the bed rest campaign without any exercise (i.e., exercise holiday), separated by a period of exercise countermeasures **(E) Rest First:** Participants start the bed rest campaign with an exercise holiday, followed by a period of performing exercise countermeasures until the end of the campaign. All of the different iterations of bed rest campaigns described above can be combined with different recovery programme modalities during post-bed rest period: Uncontrolled recovery programme: participants are not subjected to a controlled recovery programme. - Controlled but generic recovery programme: all participants complete the same recovery programme, not adapted to the personal needs of the participant. - Controlled and individualized recovery programme: all participants complete an individualize rehabilitation programme, adapted to the personal needs of the participant. Doing so may provide crucial information on the time and resources needed for an optimal recovery to take place.

It would be desirable if future HDTBR campaigns would implement durations which are directly related to the duration of future Artemis and Lunar Gateway missions, i.e., lasting anywhere between 30 and 90 days, to provide a direct implementation of the gathered knowledge to future exploration missions. Furthermore, these future bed-rest campaigns are also encouraged to explore different implementations of exercise-free periods within the duration of the campaign, as illustrated in Figure 6. Additionally, the efficacy of the different exercise devices currently on board the ISS (i.e., ARED, T2 Treadmill, CEVIS), but also the usefulness and efficacy of novel training modalities such as for example plyometric exercises (Weber et al., 2019) should be investigated to define the most optimal exercise regime and get a better understanding of rehabilitation and recovery within a (simulated) microgravity environment. Improving the definition of the optimal in-flight rehabilitation regime—which in its turn could enhance the in-flight recovery of the different physiological systems—could ultimately facilitate the acceptance of any decrements attributed to inactivity during the exercise holiday period, thus potentially increasing the time where crew would not need perform exercise countermeasures, thus enabling them to spend more time on other mission-related tasks, and ultimately to also safe critical resources (Laurens et al., 2019).

In the same way, defining the optimal recovery programme after HDTBR is warranted, as for now recovery after HDTBR is mostly uncontrolled, while crewmembers are provided—both in-flight as well as after returning to Earth—with a comprehensive and individualized exercise programme. Exploring the recovery dynamics of the different physiological systems as a result of either an uncontrolled, a controlled but generic, or a controlled and individualized recovery programme would provide crucial information on the time and resources needed for an optimal recovery to take place.

Moreover, future HDTBR campaigns should also focus on simulating upcoming Lunar Gateway mission profiles where the crew will transition from prolonged exposure to microgravity to hypogravity on the Lunar surface. Consequently, ‘conventional’ exercise stimuli such as high reaction forces and high muscle forces to stimulate bone growth (Frost, 2003), and high load resistive exercises to promote muscle hypertrophy (Yamada et al., 2012)—as is currently the case—will likely become of secondary importance. For Lunar Gateway missions with Lunar surface EVAs after a prolonged exposure to microgravity, the primary needs and requirements of the countermeasure programmes may probably undergo a shift from focussing on maintaining bone mineral density, muscle strength and VO2max to countermeasures focussing on orthostatic tolerance, postural stability, spatial orientation and balance due to the transition between microgravity and hypogravity, ultimately to assure crew safety and mission success. Such countermeasures mitigating postflight functional and sensorimotor dysfunction were also proposed by Both Miller et al. (2018) and Mulavara et al. (2018) to be incorporated in the in-flight countermeasure portfolio. The current study could be considered as the first step in exploring this potential shift in countermeasure approach as it investigated the recovery of current operationally relevant outcomes after a period of disuse, although it did not take into account the effects of countermeasures targeting postural stability, spatial orientation and balance. Therefore, future research should aim attention at further investigating this shift to aid in defining and evaluating relevant needs and requirements for in-flight countermeasures ensuring crew health and safety in upcoming space exploration missions.

Lastly, within future HDTBR studies, expansion of the current approach for standardized measurements (Sundblad and Orlov, 2014) specifically post-HDTBR is encouraged, including—but not limited to—standardization of post-HDTBR data collection (i.e., daily data collection within the first 14 days of the post-HDTBR period, followed by weekly follow-up data collection up to 3 months or longer) and standardized reporting and publishing of recovery data (i.e., reporting of raw values as means and standard deviations, the use of effect sizes, or a combination of both) thus enabling a more thorough comparison of control groups between studies, and facilitating the feasibility of retrospective analyses.

All the above would add to the body of evidence which would ultimately aid in determining whether the implementation of the concept of exercise holidays within future spaceflight operations—within and beyond LEO—would be feasible and practical.

## 5 Conclusion

The concept of exercise holidays that is presented in the current study should be regarded as one of many steps that are needed to define evidence-based needs and requirements for in-flight exercise countermeasures for future deep space exploration missions. Although a high degree of paucity and inconsistency of reported recovery data is present within the 18 included studies, data suggests that recovery of current operationally relevant outcomes following HDTBR without exercise—and even without targeted exercise rehabilitation during the recovery period—could be timely and does not lead to persistent decrements differing from those experienced following spaceflight. Thus, the concept of exercise holidays looks like a promising concept that should be further explored through space- and ground-based research to fill current knowledge gaps, prior to its potential implementation in human spaceflight exploration missions.

## Data Availability

The original contributions presented in the study are included in the article/Supplementary Material, further inquiries can be directed to the corresponding author.

## References

[B1] Alkner B. A. Norrbrand L. Tesch P. A. (2016). Neuromuscular Adaptations Following 90 Days Bed Rest with or without Resistance Exercise. Aerosp. Med. Hum. Perform. 87, 610–617. 10.3357/AMHP.4383.2016 27503040

[B2] Alkner B. r. A. Tesch P. A. (2004). Knee Extensor and Plantar Flexor Muscle Size and Function Following 90 Days of Bed Rest with or without Resistance Exercise. Eur. J. Appl. Physiol. 93, 294–305. 10.1007/s00421-004-1172-8 15338217

[B3] Arbeille P. Fomina G. Roumy J. Alferova I. Tobal N. Herault S. (2001). Adaptation of the Left Heart, Cerebral and Femoral Arteries, and Jugular and Femoral Veins during Short- and Long-Term Head-Down Tilt and Spaceflights. Eur. J. Appl. Physiology 86, 157–168. 10.1007/s004210100473 11822475

[B4] Armbrecht G. Belavý D. L. Gast U. Bongrazio M. Touby F. Beller G. (2010). Resistive Vibration Exercise Attenuates Bone and Muscle Atrophy in 56 Days of Bed Rest: Biochemical Markers of Bone Metabolism. Osteoporos. Int. 21, 597–607. 10.1007/s00198-009-0985-z 19536451

[B5] Baecker N. Tomic A. Mika C. Gotzmann A. Platen P. Gerzer R. (2003). Bone Resorption Is Induced on the Second Day of Bed Rest: Results of a Controlled Crossover Trial. J. Appl. Physiology 95, 977–982. 10.1152/japplphysiol.00264.2003 12909597

[B6] Beck L. Baisch F. Gaffney F. A. Buckey J. C. Arbeille P. Patat F. (1992). Cardiovascular Response to Lower Body Negative Pressure before, during, and after Ten Days Head-Down Tilt Bedrest. Acta Physiol. Scand. Suppl. 604, 43–52. 1509893

[B7] Belavý D. L. Beller G. Ritter Z. Felsenberg D. (2011b). Bone Structure and Density via HR-pQCT in 60d Bed-Rest, 2-years Recovery with and without Countermeasures. J. Musculoskelet. Neuronal Interact. 11, 215–226. 21885896

[B8] Belavý D. L. Beller G. Armbrecht G. Perschel F. H. Fitzner R. Bock O. (2011a). Evidence for an Additional Effect of Whole-Body Vibration above Resistive Exercise Alone in Preventing Bone Loss during Prolonged Bed Rest. Osteoporos. Int. 22, 1581–1591. 10.1007/s00198-010-1371-6 20814665

[B9] Belavý D. L. Ohshima H. Rittweger J. Felsenberg D. (2017). High-intensity Flywheel Exercise and Recovery of Atrophy after 90 Days Bed-R-est-. BMJ Open Sport Exerc. Med. 3, e000196. 10.1136/bmjsem-2016-000196 PMC5530106 28761699

[B10] Beller G. Belavý D. L. Sun L. Armbrecht G. Alexandre C. Felsenberg D. (2011). WISE-2005: Bed-Rest Induced Changes in Bone Mineral Density in Women during 60days Simulated Microgravity. Bone 49, 858–866. 10.1016/j.bone.2011.06.021 21723970

[B11] Benington S. McWilliams D. Eddleston J. Atkinson D. (2012). Exercise Testing in Survivors of Intensive Care-Is There a Role for Cardiopulmonary Exercise Testing? J. Crit. Care 27, 89–94. 10.1016/j.jcrc.2011.07.080 21958985 PMC7125548

[B12] Biering-sørensen F. Bohr H. H. Schaadt O. P. (1990). Longitudinal Study of Bone Mineral Content in the Lumbar Spine, the Forearm and the Lower Extremities after Spinal Cord Injury. Eur. J. Clin. Invest. 20, 330–335. 10.1111/j.1365-2362.1990.tb01865.x 2114994

[B13] Buckey J. C. (2006). Space Physiology. New York: Oxford University Press.

[B14] Buford T. W. Rossi S. J. Smith D. B. Warren A. J. (2007). A Comparison of Periodization Models during Nine Weeks with Equated Volume and Intensity for Strength. J. Strength Cond. Res. 21, 1245–1250. 10.1519/R-20446.1 18076234

[B15] Charles J. B. Lathers C. M. (1991). Cardiovascular Adaptation to Spaceflight. J. Clin. Pharmacol. 31, 1010–1023. 10.1002/j.1552-4604.1991.tb03665.x 1761711

[B16] Convertino V. A. Kirby C. R. Karst G. M. Goldwater D. J. (1985). Response to Muscular Exercise Following Repeated Simulated Weightlessness. Aviat. Space Environ. Med. 56, 540–546. 4015565

[B17] Convertino V. A. Doerr D. F. Eckberg D. L. Fritsch J. M. Vernikos-Danellis J. (1990). Head-down Bed Rest Impairs Vagal Baroreflex Responses and Provokes Orthostatic Hypotension. J. Appl. Physiology 68, 1458–1464. 10.1152/jappl.1990.68.4.1458 2347788

[B18] Cooke W. H. Ames J. E. Crossman A. A. Cox J. F. Kuusela T. A. Tahvanainen K. U. O. (2000). Nine Months in Space: Effects on Human Autonomic Cardiovascular Regulation. J. Appl. Physiology 89, 1039–1045. 10.1152/jappl.2000.89.3.1039 10956348

[B19] Cumming G. (2012). Understanding the New Statistics: Effect Sizes, Confidence Intervals, and Meta-Analysis. New York: Routledge/Taylor & Francis Group.

[B20] Denehy L. Elliott D. (2012). Strategies for Post ICU Rehabilitation. Curr. Opin. Crit. Care 18, 503–508. 10.1097/MCC.0b013e328357f064 22914429

[B21] Drake B. G. Hoffman S. J. Beaty D. W. (2010). Human Exploration of Mars, Design Reference Architecture 5.0. New York, NY: IEEE. 10.1109/AERO.2010.5446736

[B22] Drevon D. Fursa S. R. Malcolm A. L. (2016). Intercoder Reliability and Validity of WebPlotDigitizer in Extracting Graphed Data. Behav. Modif. 41, 323–339. 10.1177/0145445516673998 27760807

[B23] Droppert P. M. (1993). A Review of Muscle Atrophy in Microgravity and during Prolonged Bed Rest. J. Br. Interplanet. Soc. 46, 83–86. 11539498

[B24] Ferretti G. Berg H. E. Minetti A. E. Moia C. Rampichini S. Narici M. V. (2001). Maximal Instantaneous Muscular Power after Prolonged Bed Rest in Humans. J. Appl. Physiology 90, 431–435. 10.1152/jappl.2001.90.2.431 11160038

[B25] Fiebig L. Winnard A. Nasser M. Braunstein B. Scott J. Green D. (2018). Effectiveness of Resistive Exercise Countermeasures in Bed Rest to Maintain Muscle Strength and Power - A Systematic Review -. Front. Physiol. 9. 10.3389/conf.fphys.2018.26.00020

[B26] Frost H. M. (2003). Bone's Mechanostat: A 2003 Update. Anat. Rec. 275A, 1081–1101. 10.1002/ar.a.10119 14613308

[B27] Fu Q. Shibata S. Hastings J. L. Platts S. H. Hamilton D. M. Bungo M. W. (2019). Impact of Prolonged Spaceflight on Orthostatic Tolerance during Ambulation and Blood Pressure Profiles in Astronauts. Circulation 140, 729–738. 10.1161/CIRCULATIONAHA.119.041050 31319685

[B28] Gallo C. Ridolfi L. Scarsoglio S. (2020). Cardiovascular Deconditioning during Long-Term Spaceflight through Multiscale Modeling. Npj Microgravity 6, 27. 10.1038/s41526-020-00117-5 33083524 PMC7529778

[B29] Garland D. E. Stewart C. A. Adkins R. H. Hu S. S. Rosen C. Liotta F. J. (1992). Osteoporosis after Spinal Cord Injury. J. Orthop. Res. 10, 371–378. 10.1002/jor.1100100309 1569500

[B30] Gernand J. M. (2004). Risk Assessment and Control through Countermeasure System Implementation for Long-Term Crew Exposure to Microgravity. Available at: https://ntrs.nasa.gov/citations/20050220685 Accessed January 2022.

[B31] Gerstenmaier W. Crusan J. (2018). Cislunar and Gateway Overview. Available at: https://www.nasa.gov/sites/default/files/atoms/files/cislunar-update-gerstenmaier-crusan-v5a.pdf Accessed January 2022.

[B32] Green D. A. Scott J. P. R. (2018). Spinal Health during Unloading and Reloading Associated with Spaceflight. Front. Physiol. 8, 1126. 10.3389/fphys.2017.01126 29403389 PMC5778142

[B33] Greenleaf J. Quach D. (2003). Recovery after Prolonged Bed-Rest Deconditioning. Available at: https://ntrs.nasa.gov/citations/20030052739 Accessed January 2022.

[B34] Grigoriev A. I. Morukov B. V. Oganov V. S. Rakhmanov A. S. Buravkova L. B. (1992). Effect of Exercise and Bisphosphonate on Mineral Balance and Bone Density during 360 Day Antiorthostatic Hypokinesia. J. Bone Min. Res. 7, S449–S455. 10.1002/jbmr.5650071416 1485556

[B35] Hargens A. R. Richardson S. (2009). Cardiovascular Adaptations, Fluid Shifts, and Countermeasures Related to Space Flight. Respir. Physiology Neurobiol. 169, S30–S33. 10.1016/j.resp.2009.07.005 19615471

[B36] Hargens A. R. Vico L. (2016). Long-duration Bed Rest as an Analog to Microgravity. J. Appl. Physiology 120, 891–903. 10.1152/japplphysiol.00935.2015 26893033

[B37] Harris K. M. Petersen L. G. Weber T. (2020). Reviving Lower Body Negative Pressure as a Countermeasure to Prevent Pathological Vascular and Ocular Changes in Microgravity. Npj Microgravity 6, 38. 10.1038/s41526-020-00127-3 33335101 PMC7746725

[B38] Hayes J. (2015). The First Decade of ISS Exercise: Lessons Learned on Expeditions 1-25. Aerosp. Med. Hum. Perform. 86, 1–6. 10.3357/AMHP.EC01.2015 26630187

[B39] Higgins J. P. T. Thomas J. Chandler J. Cumpston M. Li T. Page M. J. (2021). Cochrane Handbook for Systematic Reviews of Interventions-Chapter 12. Chichester, United Kingdom: John Wiley & Sons. version 6.2. Cochrane Available at: www.training.cochrane.org/handbook Accessed November 2021.

[B40] Horneck G. Facius R. Reichert M. Rettberg P. Seboldt W. Manzey D. (2003). Humex, a Study on the Survivability and Adaptation of Humans to Long-Duration Exploratory Missions, Part I: Lunar Missions. Adv. Space Res. 31, 2389–2401. 10.1016/S0273-1177(03)00568-4 14696589

[B41] Kakurin L. I. Lobachik V. I. Mikhailov V. M. Senkevich Y. A. (1976). Antiorthostatic Hypokinesia as a Method of Weightlessness Simulation. Aviat. Space Environ. Med. 47, 1083–1086. 985283

[B42] Kramer A. Kümmel J. Mulder E. Gollhofer A. Frings-Meuthen P. Gruber M. (2017). High-Intensity Jump Training Is Tolerated during 60 Days of Bed Rest and Is Very Effective in Preserving Leg Power and Lean Body Mass: An Overview of the Cologne RSL Study. PLOS ONE 12, e0169793. 10.1371/journal.pone.0169793 28081223 PMC5231329

[B43] Kunz H. Quiriarte H. Simpson R. J. Ploutz-Snyder R. McMonigal K. Sams C. (2017). Alterations in Hematologic Indices during Long-Duration Spaceflight. BMC Hematol. 17, 12. 10.1186/s12878-017-0083-y 28904800 PMC5590186

[B44] Lakens D. (2013). Calculating and Reporting Effect Sizes to Facilitate Cumulative Science: a Practical Primer for T-Tests and ANOVAs. Front. Psychol. 4, 863. 10.3389/fpsyg.2013.00863 24324449 PMC3840331

[B45] Lang T. LeBlanc A. Evans H. Lu Y. Genant H. Yu A. (2004). Cortical and Trabecular Bone Mineral Loss from the Spine and Hip in Long-Duration Spaceflight. J. Bone Min. Res. 19, 1006–1012. 10.1359/JBMR.040307 15125798

[B46] Laurens C. Simon C. Vernikos J. Gauquelin-Koch G. Blanc S. Bergouignan A. (2019). Revisiting the Role of Exercise Countermeasure on the Regulation of Energy Balance during Space Flight. Front. Physiol. 10, 321. 10.3389/fphys.2019.00321 30984019 PMC6449861

[B47] LeBlanc A. Schneider V. Shackelford L. West S. Oganov V. Bakulin A. (2000). Bone Mineral and Lean Tissue Loss after Long Duration Space Flight. J. Musculoskelet. Neuronal Interact. 1, 157–160. 15758512

[B48] Leblanc A. D. Schneider V. S. Evans H. J. Engelbretson D. A. Krebs J. M. (1990). Bone Mineral Loss and Recovery after 17 Weeks of Bed Rest. J. Bone Min. Res. 5, 843–850. 10.1002/jbmr.5650050807 2239368

[B49] Levine B. D. Lane L. D. Watenpaugh D. E. Gaffney F. A. Buckey J. C. Blomqvist C. G. (1996). Maximal Exercise Performance after Adaptation to Microgravity. J. Appl. Physiology 81, 686–694. 10.1152/jappl.1996.81.2.686 8872635

[B50] Levine B. D. Zuckerman J. H. Pawelczyk J. A. (1997). Cardiac Atrophy after Bed-Rest Deconditioning. Circulation 96, 517–525. 10.1161/01.CIR.96.2.517 9244220

[B51] Linnarsson D. Spaak J. Sundblad P. (2006). Baroreflex Impairment during Rapid Posture Changes at Rest and Exercise after 120 Days of Bed Rest. Eur. J. Appl. Physiol. 96, 37–45. 10.1007/s00421-005-0062-z 16235067

[B52] Liu J. Li Y. Verheyden B. Chen Z. Wang J. Li Y. (2015). Orthostatic Intolerance Is Independent of the Degree of Autonomic Cardiovascular Adaptation after 60 Days of Head-Down Bed Rest. BioMed Res. Int. 2015, 1–10. 10.1155/2015/896372 PMC4573436 26425559

[B53] Lorenz D. S. Reiman M. P. Walker J. C. (2010). Periodization. Sports Health 2, 509–518. 10.1177/1941738110375910 23015982 PMC3438871

[B54] Matsumoto A. Storch K. J. Stolfi A. Mohler S. R. Frey M. A. Stein T. P. (2011). Weight Loss in Humans in Space. Aviat. Space Environ. Med. 82, 615–621. 10.3357/ASEM.2792.2011 21702312

[B55] Miller C. A. Kofman I. S. Brady R. R. May-Phillips T. R. Batson C. D. Lawrence E. L. (2018). Functional Task and Balance Performance in Bed Rest Subjects and Astronauts. Aerosp. Med. Hum. Perform. 89, 805–815. 10.3357/AMHP.5039.2018 30126513

[B56] Moore A. D. Downs M. E. Lee S. M. C. Feiveson A. H. Knudsen P. Ploutz-Snyder L. (2014). Peak Exercise Oxygen Uptake during and Following Long-Duration Spaceflight. J. Appl. Physiology 117, 231–238. 10.1152/japplphysiol.01251.2013 24970852

[B57] Mulavara A. P. Peters B. T. Miller C. A. Kofman I. S. Reschke M. F. Taylor L. C. (2018). Physiological and Functional Alterations after Spaceflight and Bed Rest. Med. Sci. Sports Exerc. 50, 1961–1980. 10.1249/MSS.0000000000001615 29620686 PMC6133205

[B58] NASA (2021). International Space Station Facts and Figures. Httpswwwnasagovfeaturefacts--Fig.

[B59] NASA (2020). NASA’s Lunar Exploration Program Overview. Available at: https://www.nasa.gov/sites/default/files/atoms/files/artemis_plan-20200921.pdf .

[B60] Norsk P. (2020). Adaptation of the Cardiovascular System to Weightlessness: Surprises, Paradoxes and Implications for Deep Space Missions. Acta Physiol. 228, e13434. 10.1111/apha.13434 31872965

[B61] Orwoll E. S. Adler R. A. Amin S. Binkley N. Lewiecki E. M. Petak S. M. (2013). Skeletal Health in Long-Duration Astronauts: Nature, Assessment, and Management Recommendations from the NASA Bone Summit. J. Bone Min. Res. 28, 1243–1255. 10.1002/jbmr.1948 23553962

[B62] Ouzzani M. Hammady H. Fedorowicz Z. Elmagarmid A. (2016). Rayyan-a Web and Mobile App for Systematic Reviews. Syst. Rev. 5, 210. 10.1186/s13643-016-0384-4 27919275 PMC5139140

[B63] Pavy-Le Traon A. Heer M. Narici M. V. Rittweger J. Vernikos J. (2007). From Space to Earth: Advances in Human Physiology from 20 Years of Bed Rest Studies (1986-2006). Eur. J. Appl. Physiol. 101, 143–194. 10.1007/s00421-007-0474-z 17661073

[B64] Payne M. W. C. Williams D. R. Trudel G. (2007). Space Flight Rehabilitation. Am. J. Phys. Med. Rehabil. 86, 583–591. 10.1097/PHM.0b013e31802b8d09 17167347

[B65] Perhonen M. A. Franco F. Lane L. D. Buckey J. C. Blomqvist C. G. Zerwekh J. E. (2001). Cardiac Atrophy after Bed Rest and Spaceflight. J. Appl. Physiology 91, 645–653. 10.1152/jappl.2001.91.2.645 11457776

[B66] Petersen N. Jaekel P. Rosenberger A. Weber T. Scott J. Castrucci F. (2016). Exercise in Space: The European Space Agency Approach to In-Flight Exercise Countermeasures for Long-Duration Missions on ISS. Extrem Physiol. Med. 5, 9. 10.1186/s13728-016-0050-4 27489615 PMC4971634

[B67] Petersen N. Lambrecht G. Scott J. Hirsch N. Stokes M. Mester J. (2017). Postflight Reconditioning for European Astronauts - A Case Report of Recovery after Six Months in Space. Musculoskelet. Sci. Pract. 27, S23–S31. 10.1016/j.msksp.2016.12.010 28173929

[B68] Piaggi P. Thearle M. S. Krakoff J. Votruba S. B. (2015). Higher Daily Energy Expenditure and Respiratory Quotient, rather Than Fat-free Mass, Independently Determine Greater Ad Libitum Overeating. J. Clin. Endocrinol. Metabolism 100, 3011–3020. 10.1210/jc.2015-2164 PMC4524995 26086330

[B69] Puthucheary Z. Harridge S. Hart N. (2010). Skeletal Muscle Dysfunction in Critical Care: Wasting, Weakness, and Rehabilitation Strategies. Crit. Care Med. 38, S676–S682. Available at: https://journals.lww.com/ccmjournal/Fulltext/2010/10001/Skeletal_muscle_dysfunction_in_critical_care_.26.aspx . 10.1097/CCM.0b013e3181f2458d 21164414

[B70] Quertemont E. (2011). How to Statistically Show the Absence of an Effect. Psychol. Belg. 51, 109. 10.5334/pb-51-2-109

[B71] Regnard J. Heer M. Drummer C. Norsk P. (2001). Validity of Microgravity Simulation Models on Earth. Am. J. Kidney Dis. 38, 668–674. 10.1053/ajkd.2001.27753 11532704

[B72] Rittweger J. Bareille M.-P. Clément G. Linnarsson D. Paloski W. H. Wuyts F. (2015). Short-arm Centrifugation as a Partially Effective Musculoskeletal Countermeasure during 5-day Head-Down Tilt Bed Rest-Results from the BRAG1 Study. Eur. J. Appl. Physiol. 115, 1233–1244. 10.1007/s00421-015-3120-1 25667067

[B73] Rittweger J. Felsenberg D. Maganaris C. Ferretti J. L. (2007). Vertical Jump Performance after 90 Days Bed Rest with and without Flywheel Resistive Exercise, Including a 180 Days Follow-Up. Eur. J. Appl. Physiol. 100, 427–436. 10.1007/s00421-007-0443-6 17406887

[B74] Rittweger J. Felsenberg D. (2009). Recovery of Muscle Atrophy and Bone Loss from 90 Days Bed Rest: Results from a One-Year Follow-Up. Bone 44, 214–224. 10.1016/j.bone.2008.10.044 19022418

[B75] Rittweger J. Frost H. Schiessl H. Ohshima H. Alkner B. Tesch P. (2005). Muscle Atrophy and Bone Loss after 90 Days' Bed Rest and the Effects of Flywheel Resistive Exercise and Pamidronate: Results from the LTBR Study. Bone 36, 1019–1029. 10.1016/j.bone.2004.11.014 15811637

[B76] Samel A. Wegmann H. M. Vejvoda M. (1993). Response of the Circadian System to 6 Degrees Head-Down Tilt Bed Rest. Aviat. Space Environ. Med. 64, 50–54. 8424740

[B77] Sawilowsky S. S. (2009). New Effect Size Rules of Thumb. J. Mod. App. Stat. Meth. 8, 597–599. 10.22237/jmasm/1257035100

[B78] Schulz H. Hillebrecht A. Karemaker J. M. Ten Harkel A. D. Beck L. Baisch F. (1992). Cardiopulmonary Function during 10 Days of Head-Down Tilt Bedrest. Acta Physiol. Scand. Suppl. 604, 23–32. 1509891

[B79] Scott J. P. R. Green D. A. Weerts G. Cheuvront S. N. (2020). Body Size and its Implications upon Resource Utilization during Human Space Exploration Missions. Sci. Rep. 10, 13836. 10.1038/s41598-020-70054-6 32796944 PMC7429865

[B80] Scott J. P. R. Kramer A. Petersen N. Green D. A. (2021). The Role of Long-Term Head-Down Bed Rest in Understanding Inter-individual Variation in Response to the Spaceflight Environment: A Perspective Review. Front. Physiol. 12, 9. 10.3389/fphys.2021.614619 PMC7904881 33643065

[B81] Scott J. P. R. Weber T. Green D. A. (2019). Introduction to the Frontiers Research Topic: Optimization of Exercise Countermeasures for Human Space Flight - Lessons from Terrestrial Physiology and Operational Considerations. Front. Physiol. 10. 10.3389/fphys.2019.00173 PMC6416179 30899226

[B82] Seaman M. A. Serlin R. C. (1998). Equivalence Confidence Intervals for Two-Group Comparisons of Means. Psychol. Methods 3, 403–411. 10.1037/1082-989X.3.4.403

[B83] Sibonga J. D. Evans H. J. Sung H. G. Spector E. R. Lang T. F. Oganov V. S. (2007). Recovery of Spaceflight-Induced Bone Loss: Bone Mineral Density after Long-Duration Missions as Fitted with an Exponential Function. Bone 41, 973–978. 10.1016/j.bone.2007.08.022 17931994

[B84] Smith S. M. Heer M. A. Shackelford L. C. Sibonga J. D. Ploutz-Snyder L. Zwart S. R. (2012). Benefits for Bone from Resistance Exercise and Nutrition in Long-Duration Spaceflight: Evidence from Biochemistry and Densitometry. J. Bone Min. Res. 27, 1896–1906. 10.1002/jbmr.1647 22549960

[B85] Spector E. R. Smith S. M. Sibonga J. D. (2009). Skeletal Effects of Long-Duration Head-Down Bed Rest. Aviat. Space Environ. Med. 80, A23–A28. 10.3357/ASEM.BR02.2009 19476166

[B86] Stavnichuk M. Mikolajewicz N. Corlett T. Morris M. Komarova S. V. (2020). A Systematic Review and Meta-Analysis of Bone Loss in Space Travelers. Npj Microgravity 6, 13. 10.1038/s41526-020-0103-2 32411816 PMC7200725

[B87] Stegemann J. Essfeld D. Hoffmann U. (1985). Effects of a 7-day Head-Down Tilt (-6 Degrees) on the Dynamics of Oxygen Uptake and Heart Rate Adjustment in Upright Exercise. Aviat. Space Environ. Med. 56, 410–414. 4004674

[B88] Stein T. P. Leskiw M. J. Schluter M. D. (1996). Diet and Nitrogen Metabolism during Spaceflight on the Shuttle. J. Appl. Physiology 81, 82–97. 10.1152/jappl.1996.81.1.82 8828650

[B89] Stein T. P. (2000). The Relationship between Dietary Intake, Exercise, Energy Balance and the Space Craft Environment. Pflügers Arch. - Eur. J. Physiol. 441, R21–R31. 10.1007/s004240000352 11200976

[B90] Stein T. P. (2013). Weight, Muscle and Bone Loss during Space Flight: Another Perspective. Eur. J. Appl. Physiol. 113, 2171–2181. 10.1007/s00421-012-2548-9 23192310

[B91] Sundblad P. Spaak J. Linnarsson D. (2000). Cardiovascular Responses to Upright and Supine Exercise in Humans after 6 Weeks of Head-Down Tilt (−6°). Eur. J. Appl. Physiology 83, 303–309. 10.1007/s004210000258 11138568

[B92] Sundblad P. Orlov O. (Editors) (2014). “Guidelines for Standardization of Bed Rest Studies in the Spaceflight Context,” International Academy of Astronautics. Paris: International Academy of Astronautics. Available at: https://www.nasa.gov/sites/default/files/atoms/files/bed_rest_studies_complete.pdf Accessed May 2022.

[B93] Svenningsen H. Langhorn L. Ågård A. S. Dreyer P. (2017). Post-ICU Symptoms, Consequences, and Follow-Up: an Integrative Review. Nurs. Crit. Care 22, 212–220. 10.1111/nicc.12165 25688675

[B94] Tavassoli M. (1982). Anemia of Spaceflight. Blood 60, 1059–1067. 10.1182/blood.V60.5.1059.1059 7126864

[B95] Tesch P. A. Berg H. E. Bring D. Evans H. J. LeBlanc A. D. (2005). Effects of 17-day Spaceflight on Knee Extensor Muscle Function and Size. Eur. J. Appl. Physiol. 93, 463–468. 10.1007/s00421-004-1236-9 15517339

[B96] Thornton W. E. Moore T. P. Pool S. L. (1987). Fluid Shifts in Weightlessness. Aviat. Space Environ. Med. 58, A86–A90. 3675511

[B97] Tilton F. E. Degioanni J. J. Schneider V. S. (1980). Long-term Follow-Up of Skylab Bone Demineralization. Aviat. Space Environ. Med. 51, 1209–1213. 7213266

[B98] Trappe S. Costill D. Gallagher P. Creer A. Peters J. R. Evans H. (2009). Exercise in Space: Human Skeletal Muscle after 6 Months Aboard the International Space Station. J. Appl. Physiology 106, 1159–1168. 10.1152/japplphysiol.91578.2008 19150852

[B99] Trappe T. Trappe S. Lee G. Widrick J. Fitts R. Costill D. (2006). Cardiorespiratory Responses to Physical Work during and Following 17 Days of Bed Rest and Spaceflight. J. Appl. Physiology 100, 951–957. 10.1152/japplphysiol.01083.2005 16306254

[B100] Vasilyeva T. D. Bogomolov V. V. (1991). Medical Rehabilitation Following Long-Term Space Missions. Acta Astronaut. 23, 153–156. 10.1016/0094-5765(91)90113-J 11537118

[B101] Vico L. Collet P. Guignandon A. Lafage-Proust M.-H. Thomas T. Rehailia M. (2000). Effects of Long-Term Microgravity Exposure on Cancellous and Cortical Weight-Bearing Bones of Cosmonauts. Lancet 355, 1607–1611. 10.1016/S0140-6736(00)02217-0 10821365

[B102] Watenpaugh D. E. O'Leary D. D. Schneider S. M. Lee S. M. C. Macias B. R. Tanaka K. (2007). Lower Body Negative Pressure Exercise Plus Brief Postexercise Lower Body Negative Pressure Improve Post-bed Rest Orthostatic Tolerance. J. Appl. Physiology 103, 1964–1972. 10.1152/japplphysiol.00132.2007 17947505

[B103] Waters W. W. Platts S. H. Mitchell B. M. Whitson P. A. Meck J. V. (2005). Plasma Volume Restoration with Salt Tablets and Water after Bed Rest Prevents Orthostatic Hypotension and Changes in Supine Hemodynamic and Endocrine Variables. Am. J. Physiology-Heart Circulatory Physiology 288, H839–H847. 10.1152/ajpheart.00220.2004 15486040

[B104] Waters W. W. Ziegler M. G. Meck J. V. (2002). Postspaceflight Orthostatic Hypotension Occurs Mostly in Women and Is Predicted by Low Vascular Resistance. J. Appl. Physiology 92, 586–594. 10.1152/japplphysiol.00544.2001 11796668

[B105] Weber T. Green D. A. Attias J. Sies W. Frechette A. Braunstein B. (2019). Hopping in Hypogravity-A Rationale for a Plyometric Exercise Countermeasure in Planetary Exploration Missions. PLOS ONE 14, e0211263. 10.1371/journal.pone.0211263 30759113 PMC6373893

[B106] Weber T. Scott J. P. R. Green D. A. (2020). Optimization of Exercise Countermeasures for Human Space Flight – Lessons from Terrestrial Physiology and Operational Implementation. Lausanne: Frontiers Media. Frontiers Media SA Available at: https://books.google.de/books?id=ux_UDwAAQBAJ Accessed June 2022. 10.3389/fphys.2019.01567 PMC6965165 31998142

[B107] Westby C. M. Martin D. S. Lee S. M. C. Stenger M. B. Platts S. H. (2016). Left Ventricular Remodeling during and after 60 Days of Sedentary Head-Down Bed Rest. J. Appl. Physiology 120, 956–964. 10.1152/japplphysiol.00676.2015 PMC4835908 26494448

[B108] Winnard A. Nasser M. (2017). AMSRG Bed Rest Assessment Tool v1.1.

[B109] Winnard A. Nasser M. Debuse D. Stokes M. Evetts S. Wilkinson M. (2017). Systematic Review of Countermeasures to Minimise Physiological Changes and Risk of Injury to the Lumbopelvic Area Following Long-Term Microgravity. Musculoskelet. Sci. Pract. 27, S5–S14. 10.1016/j.msksp.2016.12.009 28173932

[B110] Winnard A. Scott J. Waters N. Vance M. Caplan N. (2019). Effect of Time on Human Muscle Outcomes during Simulated Microgravity Exposure without Countermeasures-Systematic Review. Front. Physiol. 10. 10.3389/fphys.2019.01046 PMC6707384 31474878

[B111] Woolf A. D. Pfleger B. (2003). Burden of Major Musculoskeletal Conditions. Bull. World Health Organ. 81, 646–656. 14710506 PMC2572542

[B112] Yamada A. K. Verlengia R. Bueno Junior C. R. (2012). Mechanotransduction Pathways in Skeletal Muscle Hypertrophy. J. Recept. Signal Transduct. 32, 42–44. 10.3109/10799893.2011.641978 22171534

