# Supplementary material for: Introducing the Concept of Exercise Holidays for Human Spaceflight - What Can We Learn From the Recovery of Bed Rest Passive Control Groups
Source: Front Physiol. 2022 Jul 4;13:898430. doi: 10.3389/fphys.2022.898430 (PMC9307084; doi:10.3389/fphys.2022.898430)
Supplement: Supplementary file 1 [file DataSheet2.DOCX]

Supplementary Material

# Statistical Calculations

Hedges *g* with corresponding 95% Confidence Interval was calculated using the following procedure:

1. Means (M) and standard deviations (SD) of baseline measurements and follow-up time points during the recovery period were extracted.
   In case standard errors (SE) were reported instead of SD, standard deviations were calculated using the following formula^1^:

$$SD=SE* \sqrt{N}$$

with *“N”* representing the number of subjects.

In case there were only median values and interquartile ranges reported, the median was assumed to equate the mean and the SD was calculated as^2^:

$$SD=0.75*IQR$$

1. Cohen’s *d*^3^ was calculated for a within subjects design using:

$$Cohen^{'}s d=\frac{M_{post}-M_{pre}}{\sqrt{\frac{{SD}_{post}^{2}+{SD}_{pre}^{2}}{2}}}$$

with $M_{pre}$ and ${SD}_{pre}$ being the mean and standard deviation of the baseline measurement, and $M_{post}$ and ${SD}_{post}$ representing the mean and standard deviation of the measurement during a respective follow-up time point during the recovery period.

1. To account for the small sample sizes (*N* < 20), a corrected effect size, Hedges *g*^4^, was calculated

$$Hedges g=Cohen^{'}s d*\left( 1-\frac{3}{4*df-1} \right)$$

with *df* as $(N-1)$ and *“N”* the number of subjects, representing the degrees of freedom for a paired design and functioning as the standardizer.

1. Lower and Upper limits of the 95% confidence interval^5^ were calculated using

$$Hedges g \pm t_{(1-\frac{\alpha}{2},N-1)}\sqrt{\frac{2({SD}_{pre}^{2}+{SD}_{post}^{2}-2*r)}{N({SD}_{pre}^{2}+{SD}_{post}^{2})}}$$

where $t_{(1-\alpha/2,n-1)}$ refers to the *t* critical value for an alpha of 0.05 and a degree of freedom equal to $N-1$, with *“N”* the number of subjects; ${SD}_{pre}$ and ${SD}_{post}$ the standard deviations of the baseline measurement and follow-up measurement respectively; and $r$ the correlation coefficient^6^ between the baseline and follow-up scores, calculated as

$$r= \frac{d}{\sqrt{d^{2}+a}}$$

with $d$ the standardized mean difference, and a correction factor $a$ equal to 4.

# Supplementary Tables

| **Supplementary Table 1.** Keywords used for the Boolean Search String | | | | | |
| --- | --- | --- | --- | --- | --- |
| **Main category** | **Specific category** | | **Keywords in Boolean search format** | **Search number** | **Search mask** |
| Microgravity | Synonyms | | "space analogue" OR "ground-based analogue" OR "terrestrial analogue" OR "space flight" OR space-flight OR spaceflight OR "Space mission" OR "space station" OR “micro gravity” OR micro-gravity OR microgravity OR spaceflight OR weightless* OR "orbital flight" OR "zero gravity" OR "space shuttle" | 1 | Abstract/ Title |
|  | Methods & simulations | | "bed rest" OR bed-rest OR "dry immersion" OR dry-immersion | 2 | Abstract/ Title |
|  |  | | #1 AND #2 | 3 |  |
|  | Population of interest | | Astronaut* OR astronaut [Mesh] OR cosmonaut* OR taikonaut* | 4 | Abstract/ Title |
|  |  | | #1 OR #3 OR #4 | 5 |  |
| Countermeasures | Active countermeasures | | Countermeasure* OR exercis* OR exercise [Mesh] OR sport* OR "physical activity" OR "physically active" | 6 | All Fields |
|  | Passive countermeasures | | Centrifug* OR suit* OR "lower body negative pressure" OR LBNP or "fluid loading" OR garment OR stimulation OR "artificial gravity" OR "axial loading" OR electromyostimulation OR "electrical muscle stimulation" OR EMS OR "neuromuscular electrical stimulation" OR NMES OR "whole body vibration" OR WBV | 7 | All Fields |
|  | Nutritional countermeasures | | Diet, food, and nutrition [Mesh] OR nutrition* OR diet* OR food* OR supplement* OR protein* OR salt OR saline OR bi-phosphonate OR phosphonate OR nucleotide* OR vitamin* | 8 | All Fields |
|  |  | | #6 OR #7 OR #8 | 9 |  |
| Relevant parameters for mission safety | Cardiopulmonary & -vascular | Physical performance | "endurance" OR Vo2 OR Vo2max OR Vo2peak OR "maximal oxygen uptake" OR "peak oxygen uptake" OR "resting heart rate" OR "peak power" OR "maximal work load" OR "orthostatic tolerance" OR "orthostatic intolerance" OR "time until presyncope" OR "exercise tolerance" OR "central fatigue" OR "threshold" OR "onset of blood lactate accumulation" OR "OBLA" | 10 | All Fields |
|  | Biomechanical | Physical performance | "muscle strength" OR "muscular strength" OR "muscle function" OR "muscular function" OR "muscle power" OR "muscular power" OR "muscle force" OR "muscular force" OR fatigability OR "fatigue resistance" OR "peripheral fatigue" OR "joint moment" OR "joint moments" OR "postural stability" OR posture OR "postural control" OR balance OR sway OR motion OR locomotion OR gait OR walk* OR run* OR jump* OR hop* OR "movement quality" OR "movement pattern" OR "motion pattern" OR coordination OR "motor control" OR "core stability" OR "core strength" OR "trunk stability" OR "trunk strength" OR "lumbopelvic stability" OR "lumbo-pelvic stability" OR "lumbopelvic control" OR "lumbo-pelvic control" | 11 | All Fields |
|  |  | Anthropometrics | Anthropometr* OR "skeletal strength" OR "bone mineral density" OR "bone density" OR "bone mineral content" OR flexib* OR "range of movement" OR "range of motion" | 12 | All Fields |
|  |  |  | #10 OR #11 OR #12 | 13 |  |
|  |  |  | #5 AND #9 AND #13 | 14 |  |
|  |  |  | Apply human filter |  |  |

| **Supplementary Table 2.** Hedges *g* effect sizes with 95% Confidence Intervals of outcome parameters related to *‘Anthropometric Outcomes’* | | | | | | | | | |
| --- | --- | --- | --- | --- | --- | --- | --- | --- | --- |
| **Author** | **Recovery Day** | **Sample Size (n)** | **Baseline** | | **Recovery** | | **Hedges *g*** | **95% CI – Lower Limit** | **95% CI – Upper Limit** |
|  |  |  | Mean | SD | Mean | SD |  |  |  |
| *Body Mass (kg)* | | | | | | | | | |
| **Rittweger et al. 2007**^7^ | R+4 | 14 | 71.1 | 4.9 | 69.3 | 5.6 | -0.32 | -1.14 | 0.50 |
| *90 days HDTBR* | R+7 | 14 | 71.1 | 4.9 | 68.8 | 5.2 | -0.43 | -1.25 | 0.39 |
|  | R+14 | 15 | 71.1 | 4.9 | 69.7 | 5.6 | -0.25 | -1.04 | 0.53 |
|  | R+90 | 14 | 71.1 | 4.9 | 71.8 | 7.8 | 0.10 | -0.71 | 0.92 |
|  | R+180 | 15 | 71.1 | 4.9 | 72.7 | 6.1 | 0.27 | -0.51 | 1.05 |
| **Westby et al. 2016**^8^ | R+0 | 7 | 71.2 | 2.8 | 70.2 | 2.9 | -0.31 | -1.63 | 1.02 |
| *60 days HDTBR* | R+3 | 7 | 71.2 | 2.8 | 71.2 | 3 | 0.00 | -1.31 | 1.31 |
|  | R+13 | 7 | 71.2 | 2.8 | 71.4 | 3 | 0.06 | -1.25 | 1.37 |
| *BMI* | | | | | | | | | |
| **Westby et al. 2016**^8^ | R+0 | 7 | 22.6 | 0.9 | 22.3 | 0.8 | -0.31 | -1.76 | 1.15 |
| *60 days HDTBR* | R+3 | 7 | 22.6 | 0.9 | 22.6 | 0.9 | 0.00 | -1.31 | 1.31 |
|  | R+13 | 7 | 22.6 | 0.9 | 22.6 | 0.9 | 0.00 | -1.31 | 1.31 |
| **Notes.** Individual Hedges g effect sizes with 95% Confidence Interval for outcome parameters related to Anthropometric Outcomes at each reported recovery timepoint following a period of 6-degree-head-down-tilt bed rest. HDTBR: Head-down-tilt bed rest; 95% CI: 95% Confidence Interval of Hedges *g*; Kg: kilograms; BMI: Body Mass Index | | | | | | | | | |

| **Supplementary Table 3.** Hedges *g* effect sizes with 95% Confidence Intervals of outcome parameters related to the *‘Muscular System’* | | | | | | | | | |
| --- | --- | --- | --- | --- | --- | --- | --- | --- | --- |
| **Author** | **Recovery Day** | **Sample Size (n)** | **Baseline** | | **Recovery** | | **Hedges *g*** | **95% CI – Lower Limit** | **95% CI – Upper Limit** |
|  |  |  | Mean | SD | Mean | SD |  |  |  |
| *Jump Height (cm)* | | | | | | | | | |
| **Rittweger et al. 2007**^7^ | R+4 | 14 | 40.60 | 6.10 | 27.60 | 5.60 | -2.09 | -2.91 | -1.26 |
| *90 days HDTBR* | R+7 | 14 | 40.60 | 6.10 | 31.20 | 6.40 | -1.42 | -2.24 | -0.59 |
|  | R+14 | 15 | 40.60 | 6.10 | 34.10 | 5.00 | -1.10 | -1.89 | -0.31 |
|  | R+90 | 14 | 40.60 | 6.10 | 38.70 | 5.70 | -0.30 | -1.12 | 0.52 |
|  | R+180 | 15 | 40.60 | 6.10 | 39.50 | 5.80 | -0.17 | -0.96 | 0.61 |
| **Rittweger et al. 2015**^9^ | R+0 | 11 | 36.40 | 4.40 | 34.40 | 5.90 | -0.35 | -1.31 | 0.60 |
| *5 days HDTBR* | R+4 | 11 | 36.40 | 4.40 | 37.70 | 5.00 | 0.25 | -0.69 | 1.20 |
| *Peak Power (W/kg)* | | | | | | | | | |
| **Rittweger et al. 2007**^7^ | R+4 | 14 | 47.40 | 8.00 | 34.50 | 5.60 | -1.76 | -2.58 | -0.94 |
| *90 days HDTBR* | R+7 | 14 | 47.40 | 8.00 | 36.90 | 6.50 | -1.36 | -2.18 | -0.53 |
|  | R+14 | 15 | 47.40 | 8.00 | 40.20 | 7.90 | -0.86 | -1.64 | -0.07 |
|  | R+90 | 14 | 47.40 | 8.00 | 48.10 | 8.20 | 0.08 | -0.73 | 0.90 |
|  | R+180 | 15 | 47.40 | 8.00 | 48.80 | 8.40 | 0.16 | -0.62 | 0.94 |
| **Rittweger et al. 2015**^9^ | R+0 | 11 | 40.80 | 5.80 | 39.30 | 6.10 | -0.23 | -1.18 | 0.72 |
| *5 days HDTBR* | R+4 | 11 | 40.80 | 5.80 | 40.70 | 6.10 | -0.02 | -0.97 | 0.93 |
| *Supine Squat Maximal Voluntary Contraction at 90° (N)* | | | | | | | | | |
| **Alkner et al. 2016**^10^ | R+0 | 9 | 1565.00 | 386.00 | 860.00 | 260.00 | -1.93 | -3.02 | -0.85 |
| *90 days HDTBR* | R+4 | 9 | 1565.00 | 386.00 | 998.00 | 247.00 | -1.58 | -2.67 | -0.49 |
| *Supine Squat Maximal Voluntary Contraction at 120° (N)* | | | | | | | | | |
| **Alkner et al. 2016**^10^ | R+0 | 9 | 2868.00 | 929.00 | 1473.00 | 484.00 | -1.70 | -2.79 | -0.61 |
| *90 days HDTBR* | R+4 | 9 | 2868.00 | 929.00 | 1784.00 | 541.00 | -1.29 | -2.38 | -0.20 |
| *Supine Squat Concentric Peak Force (N)* | | | | | | | | | |
| **Alkner et al. 2016**^10^ | R+0 | 9 | 1537.00 | 126.00 | 923.00 | 147.00 | -4.05 | -5.14 | -2.96 |
| *90 days HDTBR* | R+4 | 9 | 1537.00 | 126.00 | 1046.00 | 208.00 | -2.58 | -3.67 | -1.49 |
| *Supine Squat Eccentric Peak Force (N)* | | | | | | | | | |
| **Alkner et al. 2016**^10^ | R+0 | 9 | 1312.00 | 232.00 | 899.00 | 160.00 | -1.87 | -2.96 | -0.78 |
| *90 days HDTBR* | R+4 | 9 | 1312.00 | 232.00 | 940.00 | 208.00 | -1.53 | -2.61 | -0.44 |
| *Supine Squat Concentric Peak Power (W)* | | | | | | | | | |
| **Alkner et al. 2016**^10^ | R+0 | 9 | 646.00 | 193.00 | 318.00 | 90.00 | -1.97 | -3.05 | -0.88 |
| *90 days HDTBR* | R+4 | 9 | 646.00 | 193.00 | 395.00 | 110.00 | -1.44 | -2.53 | -0.36 |
| *Supine Squat Work (J)* | | | | | | | | | |
| **Alkner et al. 2016**^10^ | R+0 | 9 | 462.00 | 125.00 | 276.00 | 85.00 | -1.57 | -2.66 | -0.48 |
| *90 days HDTBR* | R+4 | 9 | 462.00 | 125.00 | 395.00 | 110.00 | -0.51 | -1.60 | 0.57 |
| *Calf Press Maximal Voluntary Contraction at 90 degrees (N)* | | | | | | | | | |
| **Alkner et al. 2016**^10^ | R+0 | 9 | 2526.00 | 677.00 | 1310.00 | 519.00 | -1.82 | -2.91 | -0.73 |
| *90 days HDTBR* | R+4 | 9 | 2526.00 | 677.00 | 1502.00 | 448.00 | -1.61 | -2.70 | -0.52 |
| *Calf Press Concentric Peak Force (N)* | | | | | | | | | |
| **Alkner et al. 2016**^10^ | R+0 | 9 | 2471.00 | 576.00 | 1416.00 | 391.00 | -1.94 | -3.02 | -0.85 |
| *90 days HDTBR* | R+4 | 9 | 2471.00 | 576.00 | 1625.00 | 420.00 | -1.52 | -2.60 | -0.43 |
| *Calf Press Eccentric Peak Force (N)* | | | | | | | | | |
| **Alkner et al. 2016**^10^ | R+0 | 9 | 2442.00 | 677.00 | 1402.00 | 444.00 | -1.64 | -2.73 | -0.55 |
| *90 days HDTBR* | R+4 | 9 | 2442.00 | 677.00 | 1673.00 | 483.00 | -1.18 | -2.27 | -0.09 |
| *Calf Press Concentric Peak Power (W)* | | | | | | | | | |
| **Alkner et al. 2016**^10^ | R+0 | 9 | 470.00 | 129.00 | 206.00 | 90.00 | -2.14 | -3.23 | -1.06 |
| *90 days HDTBR* | R+4 | 9 | 470.00 | 129.00 | 258.00 | 97.00 | -1.68 | -2.76 | -0.59 |
| *Calf Press Work (J)* | | | | | | | | | |
| **Alkner et al. 2016**^10^ | R+0 | 9 | 250.00 | 50.00 | 130.00 | 47.00 | -2.23 | -3.32 | -1.15 |
| *90 days HDTBR* | R+4 | 9 | 250.00 | 50.00 | 152.00 | 47.00 | -1.82 | -2.91 | -0.74 |
| *Torque Maximal Voluntary Contraction Knee Extension at 120 degrees (Nm)* | | | | | | | | | |
| **Alkner et al. 2016**^10^ | R+1 | 9 | 218.00 | 42.00 | 87.00 | 16.00 | -3.72 | -4.81 | -2.64 |
| *90 days HDTBR* | R+10 | 9 | 218.00 | 42.00 | 135.00 | 34.00 | -1.96 | -3.05 | -0.87 |
| *Torque Maximal Voluntary Contraction Knee Extension at 90 degrees (Nm)* | | | | | | | | | |
| **Alkner et al. 2016**^10^ | R+1 | 9 | 211.00 | 23.00 | 104.00 | 22.00 | -4.29 | -5.38 | -3.21 |
| *90 days HDTBR* | R+10 | 9 | 211.00 | 23.00 | 145.00 | 34.00 | -2.05 | -3.14 | -0.97 |
| *Torque Maximal Voluntary Contraction Plantar Flexion at 90 degrees (Nm)* | | | | | | | | | |
| **Alkner et al. 2016**^10^ | R+1 | 9 | 108.00 | 22.00 | 60.00 | 15.00 | -2.30 | -3.39 | -1.21 |
| *90 days HDTBR* | R+10 | 9 | 108.00 | 22.00 | 81.00 | 14.00 | -1.32 | -2.41 | -0.23 |
| **Notes**. Individual Hedges g effect sizes with 95% Confidence Interval for outcome parameters related to the Muscular System at each reported recovery timepoint following a period of 6-degree-head-down-tilt bed rest 95% CI: 95% Confidence Interval of Hedges *g*; cm: Centimetre; W/kg: Watt per Kilogram; N: Newton; W: Watt; J: Joule; Nm: Newton-metre | | | | | | | | | |

| **Supplementary Table 4.** Hedges *g* effect sizes with 95% Confidence Intervals of outcome parameters related to the *‘Cardiovascular System’* | | | | | | | | | |
| --- | --- | --- | --- | --- | --- | --- | --- | --- | --- |
| **Author** | **Recovery Day** | **Sample Size (n)** | **Baseline** | | **Recovery** | | **Hedges *g*** | **95% CI – Lower Limit** | **95% CI – Upper Limit** |
|  |  |  | Mean | SD | Mean | SD |  |  |  |
| *Cardiac Output (l/min)* | | | | | | | | | |
| **Beck et al. 1992**^11^ | R+4 | 6 | 6.31 | 1.10 | 6.11 | 1.76 | -0.11 | -1.62 | 1.39 |
| *10 days HDTBR* | R+8 | 6 | 6.31 | 1.10 | 5.58 | 1.76 | -0.42 | -1.98 | 1.15 |
| **Westby et al. 2016**^8^ | R+0 | 7 | 5.00 | 0.40 | 5.10 | 0.30 | 0.25 | -0.21 | 0.70 |
| *60 days HDTBR* | R+3 | 7 | 5.00 | 0.40 | 5.20 | 0.40 | 0.43 | -0.50 | 1.37 |
|  | R+13 | 7 | 5.00 | 0.40 | 5.90 | 0.40 | 1.96 | -0.55 | 4.46 |
| *Heart Rate (BPM)* | | | | | | | | | |
| **Beck et al. 1992**^11^ | R+4 | 6 | 57.80 | 5.63 | 59.80 | 5.39 | 0.31 | -1.17 | 1.79 |
| *10 days HDTBR* | R+8 | 6 | 57.80 | 5.63 | 57.00 | 2.69 | -0.15 | -1.64 | 1.34 |
| **Samel et al. 1993**^12^ | R+0 | 8 | 69.00 | 19.80 | 75.00 | 14.14 | 0.31 | -0.87 | 1.49 |
| *7 days HDTBR* | R+1 | 8 | 69.00 | 19.80 | 67.00 | 16.97 | -0.10 | -1.28 | 1.09 |
| **Stegeman et al. 1985**^13^ | R+1 | 6 | 92.00 | 10.00 | 108.00 | 15.00 | 1.06 | -0.42 | 2.54 |
| *7 days HDTBR* | R+3 | 6 | 92.00 | 10.00 | 95.00 | 9.00 | 0.27 | -1.22 | 1.75 |
|  | R+5 | 6 | 92.00 | 10.00 | 92.00 | 9.00 | 0.00 | -1.48 | 1.48 |
| **Westby et al. 2016**^8^ | R+0 | 7 | 68.00 | 5.00 | 86.00 | 5.00 | 3.13 | 1.85 | 4.42 |
| *60 days HDTBR* | R+3 | 7 | 68.00 | 5.00 | 78.00 | 3.00 | 2.11 | 0.83 | 3.39 |
|  | R+13 | 7 | 68.00 | 5.00 | 77.00 | 2.00 | 2.06 | 0.78 | 3.33 |
| **Liu et al. 2015 – Fainters**^14^ | R+6 | 3 | 73.40 | 17.30 | 76.10 | 6.30 | 0.12 | -3.39 | 3.63 |
| *60 days HDTBR* | R+12 | 2 | 73.40 | 17.30 | 84.10 | 2.90 | 0.49 | -12.20 | 13.18 |
| **Liu et al. 2015 – Non-Fainters**^14^ | R+6 | 11 | 68.90 | 12.10 | 77.00 | 9.80 | 0.68 | -0.27 | 1.63 |
| *60 days HDTBR* | R+12 | 11 | 68.90 | 12.10 | 81.00 | 18.10 | 0.73 | -0.22 | 1.67 |
| *Stroke Volume (ml)* | | | | | | | | | |
| **Beck et al. 1992**^11^ | R+4 | 6 | 95.40 | 14.70 | 91.80 | 16.66 | -0.19 | -1.68 | 1.29 |
| *10 days HDTBR* | R+8 | 6 | 95.40 | 14.70 | 88.20 | 14.45 | -0.42 | -1.90 | 1.07 |
| **Westby et al. 2016**^8^ | R+0 | 7 | 72.30 | 3.80 | 61.70 | 4.30 | -2.27 | -3.61 | -0.93 |
| *60 days HDTBR* | R+3 | 7 | 72.30 | 3.80 | 66.80 | 3.30 | -1.34 | -2.68 | 0.00 |
|  | R+13 | 7 | 72.30 | 3.80 | 76.60 | 5.40 | 0.80 | -0.49 | 2.10 |
| *Left Ventricular End Systolic Volume (ml)* | | | | | | | | | |
| **Westby et al. 2016**^8^ | R+0 | 7 | 41.70 | 2.60 | 36.20 | 2.00 | -2.06 | -3.46 | -0.66 |
| *60 days HDTBR* | R+3 | 7 | 41.70 | 2.60 | 40.90 | 2.60 | -0.27 | -1.59 | 1.06 |
|  | R+13 | 7 | 41.70 | 2.60 | 42.50 | 4.20 | 0.20 | -1.10 | 1.50 |
| *Left Ventricular End Diastolic Volume (ml)* | | | | | | | | | |
| **Westby et al. 2016**^8^ | R+0 | 7 | 115.10 | 5.00 | 97.90 | 5.60 | -2.82 | -4.15 | -1.49 |
| *60 days HDTBR* | R+3 | 7 | 115.10 | 5.00 | 107.70 | 5.30 | -1.25 | -2.57 | 0.07 |
|  | R+13 | 7 | 115.10 | 5.00 | 119.00 | 8.70 | 0.48 | -0.83 | 1.78 |
| *Systolic Blood Pressure (mmHg)* | | | | | | | | | |
| **Beck et al. 1992**^11^ | R+4 | 6 | 122.50 | 11.27 | 119.20 | 12.49 | -0.23 | -1.72 | 1.25 |
| *10 days HDTBR* | R+8 | 6 | 122.50 | 11.27 | 119.80 | 2.80 | -0.28 | -1.76 | 1.21 |
| **Convertino et al. 1990**^15^ | R+2 | 11 | 118.00 | 6.63 | 118.00 | 13.27 | 0.00 | -0.95 | 0.95 |
| *30 days HDTBR* | R+5 | 11 | 118.00 | 6.63 | 118.00 | 9.95 | 0.00 | -0.95 | 0.95 |
|  | R+30 | 11 | 118.00 | 6.63 | 120.00 | 13.27 | 0.18 | -0.77 | 1.13 |
| **Westby et al. 2016**^8^ | R+0 | 7 | 114.00 | 2.00 | 114.00 | 3.00 | 0.00 | -1.31 | 1.31 |
| *60 days HDTBR* | R+3 | 7 | 114.00 | 2.00 | 114.00 | 3.00 | 0.00 | -1.31 | 1.31 |
|  | R+13 | 7 | 114.00 | 2.00 | 112.00 | 3.00 | -0.68 | -2.03 | 0.66 |
| *Diastolic Blood Pressure (mmHg)* | | | | | | | | | |
| **Beck et al. 1992**^11^ | R+4 | 6 | 56.20 | 4.90 | 55.00 | 5.88 | -0.19 | -1.67 | 1.30 |
| *10 days HDTBR* | R+8 | 6 | 56.20 | 4.90 | 55.50 | 3.92 | -0.13 | -1.62 | 1.35 |
| **Convertino et al. 1990**^15^ | R+2 | 11 | 75.00 | 9.95 | 78.00 | 9.95 | 0.28 | -0.67 | 1.23 |
| *30 days HDTBR* | R+5 | 11 | 75.00 | 9.95 | 74.00 | 6.63 | -0.11 | -1.06 | 0.84 |
|  | R+30 | 11 | 75.00 | 9.95 | 77.00 | 6.63 | 0.22 | -0.73 | 1.17 |
| **Westby et al. 2016**^8^ | R+0 | 7 | 65.00 | 2.00 | 65.00 | 2.00 | 0.00 | -1.31 | 1.31 |
| *60 days HDTBR* | R+3 | 7 | 65.00 | 2.00 | 69.00 | 3.00 | 1.36 | 0.12 | 2.61 |
|  | R+13 | 7 | 65.00 | 2.00 | 67.00 | 3.00 | 0.68 | -0.59 | 1.95 |
| *Mean Arterial Pressure (mmHg)* | | | | | | | | | |
| **Beck et al. 1992**^11^ | R+4 | 6 | 73.20 | 5.63 | 72.20 | 6.86 | -0.13 | -1.62 | 1.35 |
| *10 days HDTBR* | R+8 | 6 | 73.20 | 5.63 | 71.80 | 1.60 | -0.28 | -1.78 | 1.21 |
| **Convertino et al. 1990**^15^ | R+2 | 11 | 89.00 | 6.63 | 91.00 | 9.95 | 0.22 | -0.73 | 1.17 |
| *30 days HDTBR* | R+5 | 11 | 89.00 | 6.63 | 89.00 | 6.63 | 0.00 | -0.95 | 0.95 |
|  | R+30 | 11 | 89.00 | 6.63 | 91.00 | 6.63 | 0.28 | -0.67 | 1.23 |
| **Westby et al. 2016**^8^ | R+0 | 7 | 81.00 | 1.00 | 82.00 | 2.00 | 0.55 | -0.68 | 1.78 |
| *60 days HDTBR* | R+3 | 7 | 81.00 | 1.00 | 84.00 | 3.00 | 1.17 | -0.07 | 2.40 |
|  | R+13 | 7 | 81.00 | 1.00 | 82.00 | 2.00 | 0.55 | -0.68 | 1.78 |
| **Liu et al. 2015 – Fainters**^14^ | R+6 | 2 | 76.20 | 3.30 | 81.70 | 2.80 | 1.03 | -11.22 | 13.27 |
| *60 days HDTBR* | R+12 | 3 | 76.20 | 3.30 | 76.00 | 4.20 | -0.03 | -3.55 | 3.49 |
| **Liu et al. 2015 – Non-Fainters**^14^ | R+6 | 11 | 80.20 | 6.10 | 82.80 | 12.70 | 0.24 | -0.71 | 1.19 |
| *60 days HDTBR* | R+12 | 11 | 80.20 | 6.10 | 78.80 | 9.00 | -0.17 | -1.12 | 0.78 |
| *VO2 Peak (ml/min)* | | | | | | | | | |
| **Stegemann et al. 1985**^13^ | R+1 | 6 | 1133.00 | 120.00 | 1153.00 | 150.00 | 0.12 | -1.36 | 1.61 |
| *7 days HDTBR* | R+3 | 6 | 1133.00 | 120.00 | 1138.00 | 102.00 | 0.04 | -1.45 | 1.52 |
|  | R+5 | 6 | 1133.00 | 120.00 | 1138.00 | 154.00 | 0.03 | -1.45 | 1.51 |
| **Notes**. Individual Hedges g effect sizes with 95% Confidence Interval for outcome parameters related to the Cardiovascular System at each reported recovery timepoint following a period of 6-degree-head-down-tilt bed rest. HDTBR: Head-down-tilt bed rest95% CI: 95% Confidence Interval of Hedges *g*; l/min: Litres per Minute; BPM: Beats per Minute; ml: Millilitres; ml/min: Millilitres per Minute; | | | | | | | | | |

# References

1. Higgins, J. P. T. *et al.* *Cochrane Handbook for Systematic Reviews of Interventions - Chapter 6*. (Cochrane, 2021).

2. Hillier, S. & Inglis-Jassiem, G. Rehabilitation for Community-Dwelling People with Stroke: Home or Centre Based? a Systematic Review. *Int. J. Stroke* **5**, 178–186 (2010).

3. Goulet-Pelletier, J.-C. & Cousineau, D. A review of effect sizes and their confidence intervals, Part I: The Cohen’s d family. *Quant. Methods Psychol.* **14**, 242–265 (2018).

4. Lakens, D. Calculating and reporting effect sizes to facilitate cumulative science: a practical primer for t-tests and ANOVAs. *Front. Psychol.* **4**, 863–863 (2013).

5. Algina, J. & Keselman, H. J. Approximate Confidence Intervals for Effect Sizes. *Educ. Psychol. Meas.* **63**, 537–553 (2003).

6. Borenstein, M., Hedges, L. V., Higgins, J. P. T. & Rothstein, H. R. *Introduction to Meta-Analysis*. (John Wiley & Sons, Ltd, 2009). doi:10.1002/9780470743386.

7. Rittweger, J., Felsenberg, D., Maganaris, C. & Ferretti, J. L. Vertical jump performance after 90 days bed rest with and without flywheel resistive exercise, including a 180 days follow-up. *Eur. J. Appl. Physiol.* **100**, 427–436 (2007).

8. Westby, C. M., Martin, D. S., Lee, S. M. C., Stenger, M. B. & Platts, S. H. Left ventricular remodeling during and after 60 days of sedentary head-down bed rest. *J. Appl. Physiol.* **120**, 956–964 (2016).

9. Rittweger, J. *et al.* Short-arm centrifugation as a partially effective musculoskeletal countermeasure during 5-day head-down tilt bed rest—results from the BRAG1 study. *Eur. J. Appl. Physiol.* **115**, 1233–1244 (2015).

10. Alkner, B., Norrbrand, L. & Tesch, P. Neuromuscular Adaptations Following 90 Days Bed Rest With or Without Resistance Exercise. *Aerosp. Med. Hum. Perform.* **87**, 610–617 (2016).

11. Beck, L. *et al.* Cardiovascular Response to Lower Body Negative Pressure Before, During, and After Ten Days Head-Down Tilt Bedrest. *Acta Physiol. Scand. Suppl.* **604**, 43–52 (1992).

12. Samel, A., Wegmann, H. M. & Vejvoda, M. Response of the circadian system to 6 degrees head-down tilt bed rest. *Aviat. Space Environ. Med.* **64**, (1993).

13. Stegemann, J., Essfeld, D. & Hoffmann, U. Effects of a 7-day head-down tilt (-6 degrees) on the dynamics of oxygen uptake and heart rate adjustment in upright exercise. *Aviat. Space Environ. Med.* **56**, (1985).

14. Liu, J. *et al.* Orthostatic Intolerance Is Independent of the Degree of Autonomic Cardiovascular Adaptation after 60 Days of Head-Down Bed Rest. *BioMed Res. Int.* **2015**, 896372–896372 (2015).

15. Convertino, V. A., Doerr, D. F., Eckberg, D. L., Fritsch, J. M. & Vernikos-Danellis, J. Head-down bed rest impairs vagal baroreflex responses and provokes orthostatic hypotension. *J. Appl. Physiol.* **68**, 1458–1464 (1990).
